# Quantitative DSC Assessment of the Polymorph-Specific Crystallinity of Poly(Lactic Acid) and the Impact of a Self-Assembling Nucleating Agent and PEG Plasticizer

**DOI:** 10.3390/polym17091267

**Published:** 2025-05-06

**Authors:** Maarten Colaers, Wim Thielemans, Bart Goderis

**Affiliations:** 1 Department of Chemistry, Polymer Chemistry and Materials, KU Leuven, Celestijnenlaan 200F-Box 2404, 3001 Heverlee, Belgium; maarten.colaers@kuleuven.be; 2 Department of Chemical Engineering, Sustainable Materials Lab, KU Leuven, Campus Kulak Kortrijk, Etienne Sabbelaan 53-Box 7659, 8500 Kortrijk, Belgium; wim.thielemans@kuleuven.be

**Keywords:** poly(lactic acid), DSC, nucleating agent, plasticizer, crystallization behavior

## Abstract

This study examines the temperature-resolved, polymorph-specific crystallinity of poly(lactic acid), PLA, during cooling and heating at 10 °C/min, with a focus on the effects of N, N-bis(benzoyl) hexanedioic acid dihydrazide (BHAD, commercially known as TMC306) as nucleating agent and PEG 1000 as plasticizer. A semicrystalline (PLA-1) and amorphous (PLA-2) PLA grade were investigated. The study emphasizes the importance of using temperature-dependent, polymorph-specific transition enthalpies to accurately calculate crystallinities from Differential Scanning Calorimetry (DSC). Polymorphism is independently confirmed using Wide Angle X-ray Diffraction (WAXD). Pure PLA-1 reached an α′ crystallinity of 2% during cooling, which increased to 38% through cold crystallization upon heating. At BHAD concentrations of at least 0.4%, α crystallites formed instead of α′, reaching a maximum crystallinity of 38% during cooling. The addition of 10 wt% PEG to PLA-1 facilitated primary α crystallization during cooling, followed by secondary intraspherulitic α′ crystallization upon heating, resulting ultimately in a crystallinity of 34%. Adding 1 wt% BHAD into PLA-1 with 10 wt% PEG shifted the crystallization temperature upward by 40 °C and enhanced the α crystallinity to 44%, highlighting the synergistic effect of PEG and BHAD on crystallization.

## 1. Introduction

As environmental awareness is increasing and with upcoming regulations to promote the use of bio-based packaging materials, much attention is currently being paid to the development of bio-based, biodegradable, thermoplastic polymers such as poly(lactic acid) (PLA). PLA is an aliphatic polyester and is—besides being used in packaging—also suited for simple consumer goods [1] such as biodegradable cold drinking cups and for high-end applications such as medical scaffolds [2]. In general, PLA is a rather brittle material. Blending with other polymers or low molecular mass plasticizers can bring toughness [3]. This often goes at the expense of PLA strength and stiffness [4], unless blending also stimulates the PLA crystallinity [5].

PLA can be composed of L or D type monomers and in most cases is an L/D copolymer. The enantiomerically pure forms are referred to as PLLA or PDLA, respectively, and are crystallizable. L/D copolymers are only crystallizable when the enantiomeric purity is above 75% [6]. In addition, PLA crystals display polymorphism, with the most relevant modifications being the α and α′ forms [7]. The structure of both forms is very comparable with the latter being a more defective, less compact, and less stable version of the former. The metastable α′ form is typically grown at rather high supercooling and readily converts to the α form upon heating [7]. Blending PLLA and PDLA leads to stereocomplex crystals, which melt at higher temperatures than the crystals of pure PLLA or PDLA [8,9]. However, in almost all applications and irrespective of the enantiomeric purity, PLA remains nearly fully amorphous due to its low intrinsic crystallization rate and the high cooling rates applied during industrial processing.

Amorphous PLA displays a low heat distortion temperature [5] and poor gas barrier properties, which are not desired in packaging applications. Drieskens et al. [10] and S. F. Nassar et al. [11] demonstrated that a higher PLA crystallinity leads to better barrier properties. Similarly, M. Cocca et al. [12] found that a reduced water vapor permeability correlates with an increased PLA crystallinity. The latter authors also reported higher stiffness and strength values at higher crystallinities.

Increasing the crystallinity is thus desired and can be achieved through high temperature annealing or by the addition of a crystal nucleating agent (NA). Adding a NA is most practical from a processing point of view. Talc is a commonly used and cheap NA and is also effective in the case of PLA [5,13]. Other inorganic NAs include montmorillonite and several modified clay minerals [14]. Stereocomplex crystals can serve as organic NAs for the crystallization of pure PLLA or PDLA [15]. Other organic NAs include crystals of oligomeric aliphatic amides [16] and various types of organogelators (OG) such as sorbitol [17,18], hydrazide [19,20,21], aryl amide [22,23,24], and oxalamide [25] derivatives. These small OG molecules are most often miscible in the PLA melt and self-assemble into fine, fibrillar crystals during cooling [26]. This crystalline network possesses a high specific surface, which acts as a nucleation template for PLA crystallization. OGs are also referred to as clarifying agents as the deposition of lamellar crystallites onto the nucleating OG fibrils leads to smaller crystal aggregates than spherulites, which no longer scatter visible light. OGs are thus ideally suited to combine crystallinity with transparency, another property often desired in packaging applications.

The OG crystal network morphology and nucleating efficiency can be tuned by chemical modification [27,28], by altering the OG concentration [22], or by acting on the OG thermal history. To ensure PLA nucleation by the action of OG crystallites, one can keep the melt temperature during processing below the OG melting point [19,20,21]. However, Fan et al. [29] and Kong et al. [23] demonstrated that higher nucleation efficiencies can be reached when OG crystals are formed during cooling from a high temperature, melt-mixed state. Furthermore, Roy et al. [30] recently found out that certain oxalamide based OGs *without* epitaxial nucleating ability, are nevertheless able to nucleate PLA provided the OG solidification is induced at high supercooling. Under such conditions, the process of fine OG crystal formation exerts shear stress on the surrounding PLA melt, which triggers shear-induced PLA crystallization. This mechanism seems not to be generic because the nucleating ability of the phenylacetic acid hydrazide OG studied by Huang et al. [31] *decreased* with increasing cooling rate.

Plasticizers have also been reported to enhance PLA crystallization. They predominantly do so by increasing the chain mobility which leads to a higher crystallization rate [5,32]. This improved mobility also lowers the material glass transition temperature, $T_{g}$, by which crystallization at low temperatures is facilitated. To preserve plasticizer efficiency and material properties over the long term, plasticizer migration should be prevented. This requires compatibility with the polymer matrix.

Polyethylene glycol, PEG, is a very efficient PLA plasticizer [33]. It is nontoxic and biodegradable. F. Li et al. demonstrated that the addition of PEG to PLA leads to ductile fracture and increased crystallinity [34]. The difference in solubility parameter between PLA and PEG predicts compatibility [35]. Interestingly, when PEG is combined with a NA, a positive synergistic effect was observed on the PLA crystallization behavior, with the former enhancing the growth rate and the latter primary nucleation [4,5,24,32]. Such combinations are very relevant as they permit reaching decent crystallinities when solidified at high supercooling, such as during fast cooling or injection molding [4,5,24].

In the context of a research project targeting tough, transparent, and highly crystalline PLA with improved barrier properties, we screened a large set of commercial plasticizers for their ability to reduce the $T_{g}$, enhance the crystallinity, and minimize the plasticizer migration. In addition, the PLA crystal nucleation efficiency of different OG was tested. This screening work predominantly relied on Differential Scanning Calorimetry (DSC) to reveal how formulations affect the material crystallinity. Carefully considering polymorphism appeared to be crucial to accurately translate the subtle changes in crystallization and melting enthalpies into reliable crystallinity values.

**Figure 1:**
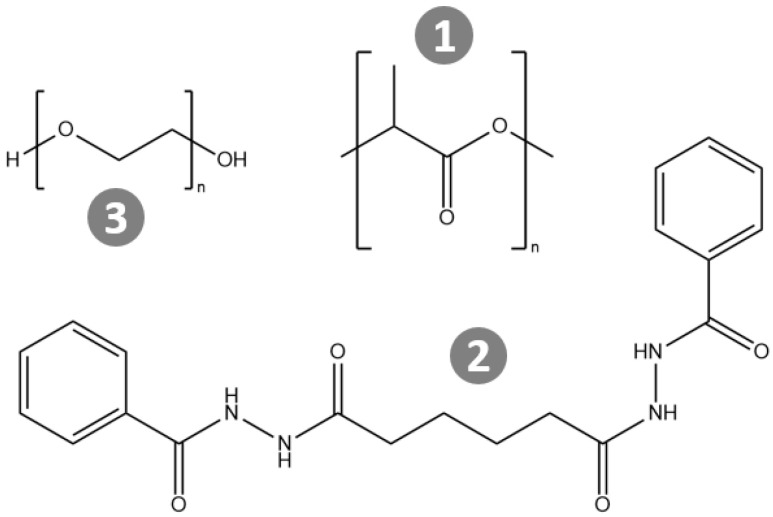
Molecular structures of the substances used in this work: (**1**) poly(lactic acid), (**2**) N, N-bis(benzoyl) hexanedioic acid dihydrazide, referred to as BHAD and commercially supplied as TMC-306 and (**3**) polyethylene glycol (with n≈23).

The current manuscript presents an in-depth analysis of the temperature-dependent crystallinity of PLA in the presence of a hydrazide OG nucleating agent and PEG 1000 as a plasticizer. The nucleating agent is N, N-bis(benzoyl) hexanedioic acid dihydrazide, referred to as BHAD and commercially available as TMC-306 from Shanxi Oriental Faith Tech Co., Ltd, Taiyuan, China (Figure 1). This combination stood out in the above-mentioned screening study and was previously investigated by Gao et al., who explored its potential to optimize PLA for fused filament fabrication [36]. Their work highlighted the synergistic effect of PEG and BHAD in enhancing PLA’s crystallization properties. Our screening study included PEG 200, PEG 400, and PEG 8000 besides PEG 1000. PEG 1000 appeared as the best option because PEG 200 and PEG 400 displayed a more pronounced leaching, in particular when PLA crystallized. The plasticizing effect of PEG 8000, as judged from its ability to reduce the PLA $T_{g}$, was slightly lower than that of PEG 1000, likely because of a lower compatibility of PEG 8000 with PLA [35].

The presented systematic and original investigation explores the effect of varying PEG and BHAD concentrations on PLA crystallinity, with a clear focus on obtaining accurate, temperature-dependent, and polymorph-specific crystallinity values from DSC. To our knowledge, no prior studies on multi-component PLA systems have offered such a detailed quantitative analysis based on DSC.

Polymorphism is independently assessed using temperature-resolved Wide Angle X-ray Diffraction (WAXD), while Polarized Optical Microscopy (POM) is employed to map the material’s morphology at the micrometer scale. Although direct crystallinity extraction from WAXD is possible, it demands measurements over a broad angular range and a precise background subtraction, which is not obvious. In contrast, DSC offers a more robust and efficient approach, provided a correct methodology is applied. Moreover, DSC allows for the analysis of larger sample sets, making it an advantageous tool for exploring crystallinity variations across different formulations.

## 2. Materials and Methods

### 2.1. Materials

Two PLA grades were provided by Nature Works LLC, Blair, Nebraska, USA: Ingeo™ Biopolymer 2500HP and Ingeo™ Biopolymer 4060D, with D-enantiomer contents of 0.3 (PLA-1) and 12% (PLA-2), respectively. Literature mentions weight average molar masses, $M_{W}$, of 190 kg/mol for both PLA-1 [37] and PLA-2 [38] and a dispersity $M_{W}/M_{N}$ of about 1.5 [39]. It will be shown that PLA-1 partially crystallizes when cooled at 10 °C/min whereas PLA-2 remains amorphous as a result of the chemical irregularity associated with the random introduction of a large fraction of D-type monomers. Amorphous PLA-2 was included because its lack of crystallinity makes it better suited for evaluating the plasticizer efficiency based on its effect on $T_{g}$, without interference from crystallization. BHAD in this work was synthesized in house based on the work of Y. Fan et al. [29] PEG (1000 g/mol) was purchased from Sigma-Aldrich. This product is crystalline at room temperature.

### 2.2. Blend Preparation

Blend films were prepared through solution casting. One gram of PLA was dissolved in 25 mL chloroform in a round-bottom flask together with appropriate amounts of additives. The flask was placed in a hot water bath at 45 °C and the solution was stirred for two hours using a magnetic stirring bar. Subsequently, the homogeneous mixture was cast into a Teflon^®^ petri dish and the solvent was left to evaporate in a fume hood. After solvent removal, the polymer films were dried in a vacuum oven at 0.1 atm and 60 °C for 16 h. For PLA with PEG, 25 °C was used instead of 60 °C. Afterwards, samples were stored in plastic zip-lock bags in a desiccator until further use. Thermogravimetric analyses showed that even under these storage conditions, the samples can contain up to 2 wt% moisture. The pure PLA reference samples received the same treatment for adequate comparison.

The BHAD content in binary PLA-1/BHAD blends was varied between 0 and 1.5 wt%. The PEG concentration in binary systems with either PLA-1 or PLA-2 was varied between 0 and 20 wt%. This content remains below the PEG solubility limit; thus, the melts are expected to be homogeneous without phase separation [5]. In ternary PLA-1/PEG/BHAD systems, PLA-1 and PEG were blended in a 90/10 PLA-1/PEG mass ratio and to this binary system a BHAD wt% varying between 0 and 1.5 wt% was added.

PLA-1 slowly crystallizes during casting, and this was found to squeeze out a fraction of the plasticizer, notwithstanding the PEG compatibility and relatively high molar mass. To determine the exact PEG wt% in the binary PLA-1/PEG blends, H^1^-NMR spectroscopy (600 Hz, Bruker) was used to quantify the plasticizer content after having performed DSC measurements on that particular sample. In Table 1, the NMR based wt% is compared to the wt% added during blending. Clearly, the PEG loss can be neglected for the 5 and 10 wt% samples but is significant for the 15 and 20 wt% PEG samples. Segregation above 10 wt% PEG 1000 was also observed by Sungsanit et al. [40]. This squeezing-out problem did not exist for PLA-2, as it remained amorphous at all times.

### 2.3. Differential Scanning Calorimetry

Differential scanning calorimetry (DSC) first heating, cooling, and second heating runs were recorded at 10 °C/min using a Q2000 heat-flux DSC (TA Instruments, Antwerp, Belgium), calibrated with an indium standard for the temperature and the enthalpy and with sapphire for the heat capacity. Open aluminum pans were used in a nitrogen atmosphere. Samples without BHAD were measured between 0 °C and 200 °C. Samples containing BHAD were characterized between 0 °C and 230 °C. Five minutes of isothermal waiting time were allowed between the heating and cooling segments. The raw heat flow data were converted into temperature-dependent specific heat signals, $c_{P}(T)$, by subtracting an empty pan signal and dividing by the sample mass and the heating or cooling rate. Next, a small linear function was subtracted to ensure that the $c_{P}(T)$ values in the liquid ($c_{pl}(T)$, Equation (1)) and in the solid state below the glass transition temperature ($c_{ps}\left(T\right)$, Equation (2)) coincided with reported reference values for pure PLA [41,42].

(1) $$c_{pl,PLA}\left(T\right)=\frac{120.7+0.076T}{72.06}$$

(2) $$c_{ps,PLA}\left(T\right)=\frac{23.33+0.2351T}{72.06}$$

In Equations (1) and (2), the temperature, T, is expressed in K and the $c_{pl,PLA(T)}$ and $c_{ps,PLA}(T)$ values in $Jg^{-1}K^{-1}$. The denominator (72.06) is the molar mass of a PLA monomeric unit in $g{mol}^{-1}$. In mixtures with PEG, the liquid reference heat capacity was calculated assuming additivity and shared according to the PEG mass fraction, $w_{PEG}$:

(3) $$c_{pl,PLA-PEG}(T)=\left(1-w_{PEG}\right)c_{pl,PLA}+w_{PEG}\left(\frac{63.95+0.07509T}{44.05}\right)$$

The factor in brackets in the second term is the reference PEG liquid heat capacity, $c_{pl,PEG}(T)$, taken from the ATHAS data base [43], where T is in K and 44.05 is the PEG monomeric molar mass in $g{mol}^{-1}$. The resulting $c_{pl,PLA-PEG}(T)$ values are expressed in $Jg^{-1}K^{-1}$. For the solid heat capacity below $T_{g}$ in blends with PEG, the PLA solid heat capacity (Equation (2)) was used because accounting for the (extrapolated) solid PEG heat capacity had a negligible impact on the value of the solid heat capacity. The contribution of small BHAD quantities to the PLA heat capacities was neglected in the definition of reference heat capacities. Finally, while determining the linear correction, an additional constraint was imposed so that the temperature-dependent transition enthalpy values, as a result of PLA crystallization and melting during cooling and subsequent heating (see Section 3), coincided at temperatures just above the glass transition temperature range in order to comply with Hess’s Law. All operations were executed by means of the Microsoft Excel Solver. Further interpretations and analyses of the $c_{p}(T)$ curves will be explained in Section 3.

When DSC data are represented as $c_{p}(T)$, both the endothermic heat associated with melting during heating and the exothermic heat from crystallization during cooling appear as positive excess heat capacities. For clarity, heating curves are plotted upward (in red) and cooling curves downward (in blue). However, the values on both sides of the $c_{p}\left(T\right)=0$ axis remain positive. $c_{p}(T)$ values are negative only when the exothermic heat released during cold crystallization upon heating causes the signal to dip below the $c_{p}\left(T\right)=0$ line.

### 2.4. Synchrotron Wide Angle X-Ray Diffraction

Time-resolved Wide Angle X-ray Diffraction (WAXD) patterns were collected at BM26, the Belgian beamline for small and wide angle scattering at the European Synchrotron Radiation Facility (ESRF, Grenoble, France). A DSC600 hotstage coupled to a T95-LINK PC interface controller and an LNP95 cooling system (Linkam Scientific Instruments, Redhill, UK) was used for temperature control. The samples, wrapped in aluminum foil, were cooled from the melt to 25 °C at 10 °C/min and after 1 min heated again to 200 °C at the same rate. Initial melt temperatures are sample dependent and were selected as in DSC. WAXD patterns were collected on a Pilatus 300K detector (Dectris, Baden, Switzerland) in consecutive 6 s time frames, leading to a WAXD pattern every °C. The scattering angles were calibrated using the 110 and 200 reflections of high density polyethylene, cooled at 10 °C min^−1^. A wavelength (λ) of 1.033 Å (12 keV) was used. After azimuthally averaging the 2D WAXD patterns using the Conex 1.5 software [44], the linear WAXD powder patterns were normalized to the intensity of the incoming beam and corrected for the background by subtracting the pattern of an aluminum foil, taking into account transmission differences. The WAXD intensities are expressed as a function of the scattering angle, 2θ. To facilitate comparison with literature data, equivalent scattering angles were adopted as if ${Cu}_{Κ\alpha }$ radiation (*λ* = 1.54 Å) was used. Data of selected samples are discussed over the angular range $14{}^{\circ}\leq 2\theta_{{Cu}_{K\alpha }}\leq 20{}^{\circ}$.

### 2.5. Optical Microscopy

Time-resolved (polarized) optical microscopy was used to follow the crystallization behavior under DSC-like thermal conditions. To this end, an Olympus BH2 microscope was equipped with a ProgRes^®^ CF camera (Jenoptik Optical Systems GmBH, Monheim am Rhein, Germany), and images were captured using the ProgRes^®^ CapturePro 2.10.0.1 software from Jenoptik. A small amount of sample was melt pressed between two glass cover slips and the temperature was controlled using a Linkam optical DSC600 Hotstage, with a Linkam TMS 91 control unit.

## 3. Results and Discussion

This section is divided into four subsections. Section 3.1 discusses the thermal behavior of the pure polymers. In Section 3.2, binary blends of PLA-1 with the OG BHAD are discussed and in Section 3.3, binary blends of PLA-1 or PLA-2 with the plasticizer PEG are treated. In Section 3.4, ternary PLA1-BHAD-PEG systems are discussed.

### 3.1. Pure PLA

**Figure 2:**
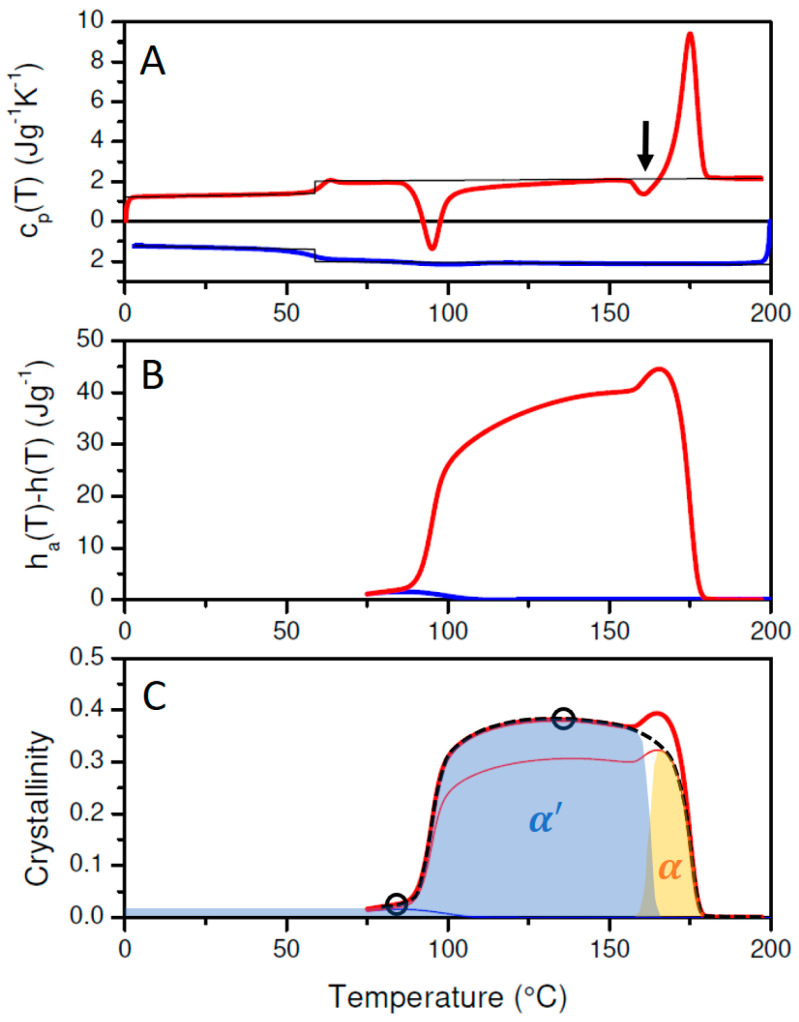
(**A**): DSC cooling (blue) and subsequent heating (red) run on PLA-1 at 10 °C/min between 200 and 0 °C at 10 °C/min. The thin black curves are reference $c_{p}\left(T\right)$ values for amorphous PLA, given by Equations (1) and (2), with a changeover at the inflection point based $T_{g}$. The arrow points at the exothermic conversion from the α′ to the α phase. (**B**): transition enthalpy values during cooling (blue) and heating (red), derived from the $c_{p}\left(T\right)$ values; (**C**): $c_{p}\left(T\right)$ based crystallinity values, assuming α′ (thick full lines) or α (thin full lines) crystallinity during cooling (blue) and heating (red). The dashed black curve represents the overall crystallinity evolution during heating, interpreted as a superposition of α′ (blue area) and α (yellow area) crystallinity. The circled points highlight the maximum α′ crystallinity values during cooling (at 87 °C) and heating (at 134 °C).

The curves in Figure 2A show the $c_{p}(T)$ data of solution cast PLA-1 during cooling (blue curve) from 200 to 0 °C at 10 °C/min and subsequent reheating at the same rate (red curve). The thin black lines are the reference heat capacity values given by Equations (1) and (2). The changeover from $c_{pl,PLA(T)}$ to $c_{ps,PLA}$ occurs at the glass transition temperature, $T_{g}$, given by the inflection point at the $c_{p}(T)$ jump.

The DSC-based temperature-dependent mass fraction crystallinity, $w_{c}(T)$, was calculated using [45]:

(4) $$w_{c}\left(T\right)=\frac{h_{a}\left(T\right)-h(T)}{h_{a}\left(T\right)-h_{c}(T)}=\frac{h_{a}\left(T\right)-h(T)}{∆h_{ref}(T)}$$

with $h_{a}\left(T\right)$ and $h_{c}(T)$ representing the specific enthalpy of fully amorphous and fully crystalline PLA, respectively, and h(T) representing the (experimental) specific enthalpy of semicrystalline PLA. In practice, the numerator of Equation (4) for temperatures above $T_{g}$ was obtained via [45]:

(5) $$h_{a}\left(T\right)-h\left(T\right)=\frac{\left[h_{a}\left(T_{ref}\right)-\int_{T_{ref}}^{T}c_{pl,PLA}\left(T\right)dT\right]-\left[h_{a}\left(T_{ref}\right)-\int_{T_{ref}}^{T}c_{p}\left(T\right)dT\right]}{w_{PLA}}\;=\frac{1}{w_{PLA}}\int_{T_{ref}}^{T}\left[c_{p}\left(T\right)-c_{pl,PLA}\left(T\right)\right]dT$$

$T_{ref}$ is a reference temperature in the melt state, which was arbitrarily set to 170 °C during cooling and 195 °C during heating in this study. Formally, to obtain $h_{a}\left(T\right)$ or $h\left(T\right)$ (given by the first and second bracketed terms, respectively, in Equation (5)), one needs to know the absolute specific enthalpy at the reference temperature, $h_{a}\left(T_{ref}\right)$, but upon subtracting $h\left(T\right)$ from $h_{a}\left(T\right)$, this parameter vanishes (second line of Equation (5)). The PLA mass fraction, $w_{PLA}$, in this equation directs the calculation to PLA in the case of the blends with PEG, as discussed in the following sections. Figure 2B illustrates the evolution of the numerator in Equation (4) for PLA-1 during cooling (blue curve) and subsequent heating (red curve). This function corresponds to the temperature-dependent transition enthalpy to convert the semicrystalline state to the amorphous liquid. Dividing this experimental transition enthalpy by the enthalpy change associated with a conversion of a fully crystalline state, $∆h_{ref}(T)$, yields the material crystallinity (Equation (4)). However, $∆h_{ref}$ depends on the crystal modification. The relevant modifications are the α and α′ forms [7], for which the respective reference transition enthalpies, $∆h_{ref,\alpha }(T)$ and $∆h_{ref,\alpha^{\prime }}(T)$ were derived by Righetti et al. (with T in °C) [46]:

(6) $$∆h_{ref,\alpha^{\prime }}\left(T\right)=20.9+0.74T-0.0011T^{2}$$

(7) $$∆h_{ref,\alpha }\left(T\right)=45.7+0.74T-0.0011T^{2}$$

$∆h_{ref,\alpha }\left(T\right)$ is larger than $∆h_{ref,\alpha^{\prime }}\left(T\right)$ because the enthalpy of the most stable α crystals is lower than that of the metastable α′ crystals. To obtain the Equations (6) and (7), experimental melting enthalpies were calibrated against WAXD crystallinities [46]. More recently, alternative reference transition enthalpies were proposed, in which the calibration relied on the crystallinity dependent specific heat capacity at a temperature slightly higher than the glass transition temperature and the conviction that no rigid amorphous fraction (RAF) exists [47,48]. Although many interesting arguments are provided for the absence of RAF [47], more research seems to be required to align this notion with reports stating that RAF gradually develops already at temperatures below 140 °C [49]. Secondly, crystallinity values from data discussed in the current manuscript, based on these alternative reference enthalpies occasionally reached 70%. This value, also reported by Jariyavidyanont et al. [47], is exceptionally high and not confirmed by WAXD. Therefore, it was decided to make use of the earlier WAXD calibrated estimates, i.e., the Equations (6) and (7).

Figure 2C displays $w_{c}(T)$ for PLA-1, calculated assuming α′ (Equation (6)—thick blue and red lines) or α (Equation (7)—thin lines) crystallinity. Following Zhang et al., the α′ modification is generated when crystallized at *T* < 100 °C, whereas the α form dominates at *T* > 120 °C [7]. Mixed forms occur when crystallized in the region 100–120 °C. Given that crystallization is only triggered below 100 °C during cooling, and that cold crystallization is almost finished before reaching 120 °C (Figure 2A), it seems that PLA-1 reaches a maximum α′ crystallinity of 2.0% at 69 °C during cooling and a maximum α′ crystallinity at 130 °C of 38.2% during heating. These values are highlighted using open black circles in Figure 2C.

Upon further heating, an exothermic signal appears just before the main melting peak (marked by the black arrow in Figure 2A), which has been assigned to a transition from the α′ to the α phase [7]. At the highest temperatures within the final melting peak, only α crystals are supposed to be left [7], and the actual crystallinity should be given by the red thin α curve rather than by the red thick α′ curve. So, as the PLA-1 crystallinity is of α′ nature at low and of α nature at high temperatures, the actual PLA-1 crystallinity should swap from the α′ to the α crystallinity line in Figure 2. This expected overall crystallinity evolution is indicated in Figure 2C by the dashed black curve. This crystallinity is to be interpreted as the sum of the declining α′ crystallinity (blue shaded area) and the increasing α crystallinity (yellow shaded area). This interpretation in terms of a polymorphic transition is fully compatible with the overall transition enthalpy evolution (Figure 2B). Indeed, the change due to this transition, ${∆h}_{\alpha^{\prime }\to \alpha }$, from the temperature at the onset of the transition ($T_{1}$) to its offset ($T_{2}$), can be calculated from:

(8) $${∆h}_{\alpha^{\prime }\to \alpha }=\left[{w_{c,\alpha^{\prime }}\left(T_{2}\right)\cdot ∆h_{ref,\alpha^{\prime }}\left(T_{2}\right)-w}_{c,\alpha^{\prime }}\left(T_{1}\right)\cdot ∆h_{ref,\alpha^{\prime }}\left(T_{1}\right)\right]+\left[w_{c,\alpha }\left(T_{2}\right)\cdot ∆h_{ref,\alpha }\left(T_{2}\right)-w_{c,\alpha }\left(T_{1}\right)\cdot ∆h_{ref,\alpha }\left(T_{1}\right)\right]$$

with $w_{c,\alpha^{\prime }}(T)$ and $w_{c,\alpha }(T)$ representing the α′ and α mass fraction crystallinities, respectively, and $∆h_{ref,\alpha^{\prime }}\left(T\right)$ and $∆h_{ref,\alpha }\left(T\right)$ given by the Equations (6) and (7). In this specific case, $w_{c,\alpha^{\prime }}\left(T_{2}\right)$ and $w_{c,\alpha }\left(T_{1}\right)$ are zero. With $T_{1}$ and $T_{2}$ being 153.6 and 166.8 °C, respectively, and $w_{c,\alpha^{\prime }}(T)$ and $w_{c,\alpha }(T)$ being 0.369 and 0.319, respectively, this leads to ${∆h}_{\alpha^{\prime }\to \alpha }=4.15\;Jg^{-1}$, which is equal to the upward step in Figure 2B or the area of the exothermic conversion signal in Figure 2A (indicated by the black arrow). It should be clear that interpretations in terms of only α′ or α (the red and blue curves in Figure 2C) are in fact equally compatible with the DSC data. However, in these cases, the exothermic signal at the arrow in Figure 2A is interpreted as an increase in the α′ or α crystallinity.

**Figure 3:**
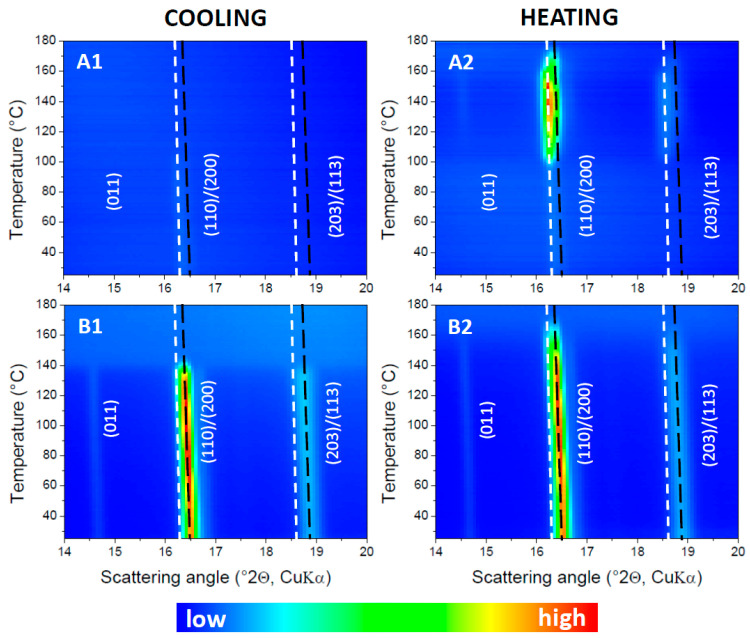
WAXD patterns during cooling (left side **A1**,**B1** panels) and subsequent heating (right side **A2**,**B2** panels) at 10 °C/min for pure PLA-1 (top **A**-panels) and PLA-1 with 1 wt% BHAD (bottom **B**-panels). The patterns are top viewed with color coded intensities from low (blue) to high (red) intensities according to the color scale at the bottom. Numbers in between brackets are Miller indices of pertinent reflections. The dashed lines mark out the tracks of the mixed (110)/(200) and (203)/(113) reflections expected for the α′ phase in white and for the α phase in black [7].

The interpretation in terms of an α′ to α transition is corroborated by the temperature-resolved WAXD data, which are displayed in Figure 3(A1) for the cooling and in Figure 3(A2) for the heating run. In these Figures, the dashed lines mark out the tracks of the mixed (110)/(200) and (203)/(113) reflections expected for the α′ phase in white and for the α phase in black, following Zhang et al. [7]. Close inspection reveals that α′ type reflections are generated during cooling from approximately 100 °C onwards. The intensity remains very low (and hardly visible in Figure 3(A1)) down to 25 °C, as expected, given that only 2.0% crystallinity is reached according to DSC. This remains so during subsequent heating up to 100 °C where the α′ type crystallinity increases strongly by cold crystallization. The grown α′ reflections start to reduce at about 150 °C, while at the same time α type reflections appear. Indeed, in that temperature range during heating, the scattered intensities leave the white dashed α′ track and start coinciding with the black dashed α track in Figure 3(A1). When reaching roughly 160 °C, the α′ reflections are gone while α type reflections remain, which ultimately disappear by complete melting at approximately 175 °C.

**Figure 4:**
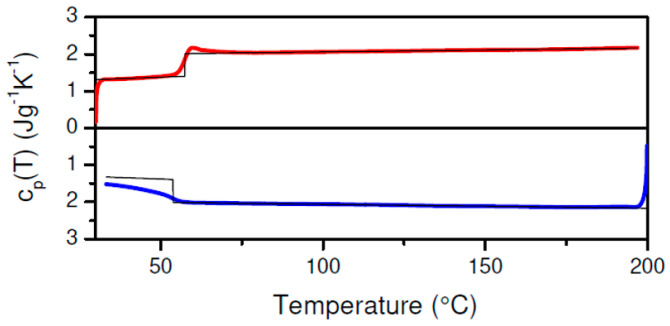
DSC cooling (blue) and subsequent heating (red) run on PLA-2 at 10 °C/min between 200 and 0 °C at 10 °C/min. The thin black curves are reference $c_{p}\left(T\right)$ values for amorphous PLA given by the Equations (1) and (2), with a changeover at the inflection point based $T_{g}$.

PLA-2 during cooling and heating at 10 °C/min remained fully amorphous and only displays a glass transition in Figure 4. The $c_{p}\left(T\right)$ inflection point based PLA-2 $T_{g}$ at 53.9 °C during cooling is lower than that of PLA-1 at 57.7 °C. This difference is due to the higher D-enantiomer content in PLA-2, given that the molar mass parameters for the two materials are comparable [50]. In principle, the material crystallinity can lift up $T_{g}$ but as PLA-1 crystallinity during cooling only reached 2.0%, this effect should be limited. In any case, the $T_{g}$ values obtained here compare well with values from a study in which the impact of the D-enantiomer content on the PLA $T_{g}$ was addressed [51].

### 3.2. Binary Blends of PLA-1 with BHAD

**Figure 5:**
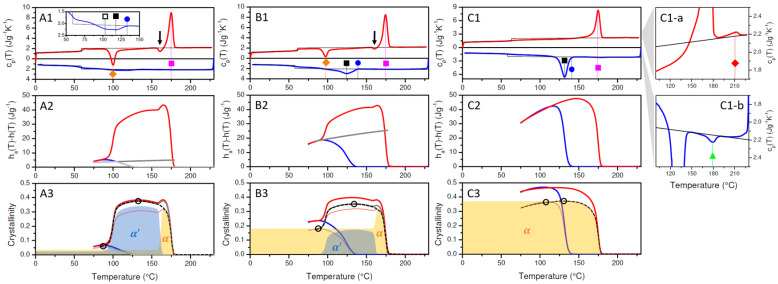
DSC measurement of PLA-1 with 0.25 (**A**), 0.35 (**B**), and 1.00 (**C**) w% BHAD during cooling (blue curves) and subsequent heating (red curves). The panels 1, 2, and 3 cover the $c_{p}(T)$, transition enthalpy, and crystallinity, respectively (thick and thin lines for treatments in terms of α′ and α crystals, respectively). The thin black curves in panel 1 are reference $c_{p}\left(T\right)$ values for amorphous PLA given by the Equations (1) and (2), with a changeover at the inflection point based $T_{g}$. The arrows in the panels (**A1**,**B1**) point at the exothermic transition associated with a conversion from the α′ to the α phase. The panels (**C1-a**,**C1-b**) are magnifications of panel (**C1**) to highlight the crystallization (in **C1-b**) and melting (in **C1-b**) peaks of BHAD. Panel **A1** also contains a magnification of the cooling run for the sample with 0.25 wt% BHAD. Certain peak and onset temperatures of thermal transitions are highlighted using vertical lines with symbols: $T_{c,NA}$ (green triangles), $T_{co,PLA}$ (blue circles), $T_{cp,PLA}$ (black squares, open: not nucleated by BHAD, closed: nucleated by BHAD), $T_{ccp,PLA}$ (orange diamond), $T_{m,PLA}$ (magenta squares), and $T_{m,NA}$ (red diamonds). The grey line in the panels (**A2**,**B2**) are calculated transition enthalpies, assuming a constant α crystallinity at the level reached during preceding cooling. The dashed black curve in the panels (**A3**,**B3**,**C3**) represent the overall crystallinity evolution during heating, which is to be interpreted as a superposition of α′ (blue area) and α (yellow area) crystallinity. The thin (for the cooling run) and thick (for the heating run) grey curves in the panels (**A3**,**B3**) illustrate evolutions of mixed of α′ and α crystallinities. The circled data points highlight the maximum crystallinity values reached during cooling and heating.

The BHAD content in this study was varied between 0 and 1.5 wt% in PLA-1, i.e., 0, 0.15, 0.25, 0.35, 0.50, 1, and 1.5 wt% BHAD. In Figure 5, the DSC result of three representative PLA-1/BHAD blends are shown: 0.25 wt% BHAD in Figure 5(A1–A3), 0.35 wt% in Figure 5(B1–B3), and 1 wt% in Figure 5(C1–C3). The $c_{p}(T)$ data were treated the same as for pure PLA-1. However, for the blends, the specific transition enthalpies were normalized to the actual PLA-1 mass fraction in the sample ($w_{PLA}$ in Equation (5)). Therefore, all enthalpy and crystallinity values relate to the PLA mass only. This approach was used for all PLA-1 blends in this work.

Crystallization is clearly stimulated by the addition of BHAD. When 1 wt% BHAD is added, crystallization during cooling is pronounced and no cold crystallization is observed during subsequent heating (Figure 5(C1)). As crystallization during cooling is essentially over prior to reaching 120 °C, it can be inferred that all crystals should be of the α form [7]. This is confirmed in the corresponding WAXD patterns displayed in the Figure 3B where all diagnostic reflections follow the α phase tracks, i.e., the black dashed lines, during cooling as well as during subsequent heating. Also, in the DSC heating run, the absence of α′ crystals is evident as no exothermic signal prior to the main melting peak is observed, which would have revealed the presence of α′ crystals through their conversion into α species. Crystallinities were calculated and represented in Figure 5(C3) assuming α′ (thick lines) or α crystals (thin lines). Given the foregoing discussion, only the α curves are relevant in this case with 1 wt% BHAD. For that reason, the overall crystallinity during heating (the dashed black curve in Figure 5(C3)) is shaded yellow over the entire track. On similar grounds, it was concluded that pure α crystals are formed also in the samples with 0.5 and 1.5 wt% BHAD.

**Figure 6:**
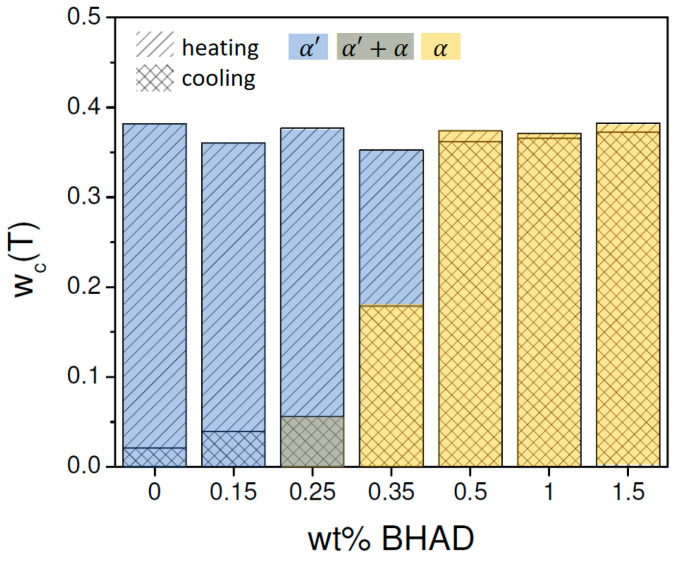
Mass fraction crystallinities reached during cooling at 10 °C/min (crosshatched parts of the bars) and the further increase realized during cold crystallization in the subsequent heating run at the same rate (diagonally hatched parts of the bars) for PLA-1 as function of the BHAD content (wt%). The total bar height represents the sum of contributions during cooling and heating. The crystal form (α′ in blue, α in yellow, and mixed forms in greenish shading) is indicated. This Figure contains the data marked with the open black circles in the Figure 2 and Figure 5.

Note that the crystallinity curves in Figure 5(C3) tend to decline towards the lowest temperatures, as if the crystallinity would decrease during cooling after having passed 120 °C. This is an artifact of neglecting the formation of a rigid amorphous fraction (RAF), which vitrifies prior to reaching the normal bulk $T_{g}$, as a result of being connected to the PLA crystals [49]. This effect is larger the higher the crystallinity and leads to a $c_{p}\left(T\right)$ reduction, which translates into an apparent crystallinity reduction in treatments that assume a simple (crystalline-liquid) two-phase system [45]. As this is an artifact, the yellow shading, indicating the α crystalline fraction, was extended down to the lowest temperatures using the highest crystallinity value reached, i.e., 0.37. The maximum α crystallinity reached during cooling amounts to 0.36 at 109 °C but is slightly higher during heating, i.e., 0.37 at 132.6 °C. These values are highlighted using open black circles in Figure 5(C3) and are compared with crystallinity values for other BHAD contents in Figure 6. Very likely, this (small) difference between the cooling and heating crystallinity values, which also is observed for PLA-1 with 0.5 and 1.5 wt% BHAD (Figure 6), is also related to RAF formation, which more strongly affects the crystallinity at lower temperatures. In other words, as these differences are not real, crystallization in PLA-1 systems with at least 0.5 wt% BHAD is completed during cooling and not augmented further during subsequent heating.

At first sight, the crystallization behavior of PLA-1 with 0.35 wt% BHAD (Figure 5B) looks similar to that of pure PLA-1 (Figure 2), except that a higher crystallinity is reached during cooling. However, as the crystallization onset during cooling occurs at 138 °C and as the crystallization peak does not stretch below 100 °C, most—if not all—crystals should be of the α form, just like for the samples with a higher BHAD content. On the other hand, as cold crystallization is triggered far below 120 °C (Figure 5(B1)), crystals formed at that transition should be of the α′ form. Moreover, the cold crystallization signal in fact coincides in temperature with that of pure PLA-1, suggesting that this part of the crystallization happens just like in pure PLA-1, i.e., by homogeneous nucleation, independently from BHAD. The creation of α crystals during cooling in the 0.35 wt% BHAD sample can also be inferred from the heating run. The exothermic peak just before the final melting endotherm in Figure 5(B1) (as indicated with an arrow) points at a conversion from α′ to α crystals but the magnitude of this transition (integration leads to $1.10\;Jg^{-1}$) is significantly lower than the value obtained for pure PLA-1 ($4.15\;Jg^{-1}$), suggesting that less α′ crystals are present to convert to α crystals. At the start of heating, the 0.35 wt% BHAD sample thus contains α crystals, the amount of which is given by the maximum α crystallinity reached during cooling, i.e., 0.18. This level is shaded yellow in Figure 5(B3) and is constant until it increases as a result of recrystallization from the α′ phase. The transition enthalpy for that constant α fraction was computed as $0.18\cdot ∆h_{ref,\alpha }\left(T\right)$ and is the grey line in Figure 5(B2). The transition enthalpy obtained after subtracting this share from the total transition enthalpy was interpreted in terms of α′ crystallinity. The sum of those crystalline fractions is represented by the grey curve in Figure 5(B3) and logically falls in between the calculations that assume pure α or α′ crystallinity. The actual crystallinity evolution during heating, given by the black dashed curve in Figure 5(B3), runs over the grey curve with mixed α and α′ crystallinities up to when it transfers to the pure α crystallinity curve in the α′ − α transition temperature range. The actual α′ and α shares to the overall crystallinity during heating are shaded in blue and yellow in Figure 5(B3), respectively. The enthalpy associated with the α′ − α transition can be computed with Equation (8). Note that in this case $w_{c,\alpha }\left(T_{1}\right)$ equals 0.18, rather than zero. The result is $1.10\;Jg^{-1}$, as expected. Again, the two circled data in Figure 5(B3) mark out the maximum crystallinity levels reached during cooling and heating, which are also included in Figure 6.

Cooling the 0.25 wt% BHAD sample leads to a bimodal solidification behavior as the exothermic signal displays two maxima (Figure 5(A1) and its inset). The low temperature peak (highlighted with open square) occurs at approximately 103 °C, which is very close to the crystallization peak for pure PLA-1 (99.5 °C), suggesting a common origin, i.e., the creation of α′ crystals through spontaneous nucleation. The high temperature peak at 116.2 °C (marked with a black square), with an onset at 132.5 °C (marked with blue circle) is interpreted as BHAD induced α crystallization, like in the 0.35 wt% BHAD sample. Cold crystallization during heating again occurs at very low temperatures where α′ crystals are expected to sprout through homogeneous nucleation. Upon heating, part of the α′ crystals convert into α crystals prior to final melting, as can be deduced from the exothermic signal before the main melting peak and as indicated with an arrow in Figure 5(A1).

Splitting the bimodal cooling exothermic signal for the sample with 0.25 wt% BHAD into α and α′ shares is a bit ambiguous. In this case, α crystals were assumed to grow at the high temperature side of the exotherm up to a crystallinity level that leaves space for as much α′ crystallinity as in pure PLA-1 during cooling (0.02). In practice, this leads to a maximum α crystallinity during cooling of 0.035. The transition enthalpy up to this level of α crystallinity during cooling is represented using the thin grey curve in Figure 5B. The thick grey curve represents the constant α transition enthalpy at the 0.035 crystallinity level during subsequent heating. The transition enthalpy above these grey levels was attributed to α′ crystallinity. The result of this mixed crystallinity evaluation is shown in Figure 5(A3), using thin and thick grey lines for the cooling and heating runs, respectively. The overall crystallinity follows these grey curves up to when reaching the α′ − α transition temperature range during heating, where it shifts to the α crystallinity line. The overall crystallinity during heating is covered with the dashed black curve in Figure 5(A3) and should be interpreted as a sum of α (shaded in yellow) and α′ (shaded in blue) crystallinity. The maximum crystallinity values reached during cooling and heating are highlighted with open circles and included in Figure 6.

For the sample with 0.15 wt% BHAD, the amount of BHAD nucleated α crystals must be very small, as no bimodal crystallization behavior during cooling was observed. Therefore, the crystallinity of this sample was analyzed by neglecting a potential α share and assuming α′ crystallinity throughout, with an α′ − α transition prior to full melting. The maximum α′ crystallinity values during cooling and heating are included in Figure 6. The crystallinity of the 0.15 wt% sample during cooling is a little higher than that of pure PLA-1, suggesting a minor nucleating activity. The input for pure PLA-1 in Figure 6 is highlighted with open circles in Figure 2C.

Clearly, BHAD addition stimulates crystallization during cooling and reaches a maximum at 0.5 wt% BHAD or beyond (Figure 6). Furthermore, at this stage, all crystals are of the α form rather than of the α′ form when cooled at 10 °C/min. Interestingly, the total mass fraction crystallinity as a result of crystallization during cooling together with cold crystallization during heating seems to be independent of the BHAD wt% (Figure 6), implying that the maximum crystallinity does not depend on the actual crystallization temperatures (which are different for the different BHAD contents) or the crystal form.

This conclusion relies on a proper use of the reference transition enthalpies, $∆h_{ref,\alpha }(T)$ and $∆h_{ref,\alpha^{\prime }}(T)$. If $∆h_{ref,\alpha^{\prime }}(T)$ would have been used instead of $∆h_{ref,\alpha }(T)$ for the samples with more than 0.35 wt% BHAD, crystalline mass fractions would have amounted to 0.46 rather than to 0.37 because $∆h_{ref,\alpha^{\prime }}(T)<∆h_{ref,\alpha }(T)$. It is also relevant to emphasize that using temperature-dependent reference transition enthalpies is crucial to reach correct conclusions. Classical integration of the exothermic peak during cooling in Figure 5(B1) (using a melt extrapolated baseline), leads to a transition enthalpy of 42.3 J g^−1^ for the sample with 1 wt% BHAD, whereas integration of the endothermic peak during heating leads to 46.7 J g^−1^. Carelessly dividing these values by 93.1 J g^−1^ (an often cited value for the enthalpy of 100% crystalline PLA [4,5,51]) would lead to a crystalline mass fraction of 0.45 during cooling and of 0.50 during heating, which could easily—but erroneously—be interpreted in terms of a (hidden) cold crystallization or a crystal stability enhancement. With a correct temperature-dependent analysis, rather equal maximum crystallinities during cooling and heating are obtained for the 1 wt% BHAD sample, i.e., approximately 0.37 as shown in Figure 6. Note that the classically obtained crystalline fractions (0.45 and 0.50) are also much larger than the 0.37 reported in the current work.

**Figure 7:**
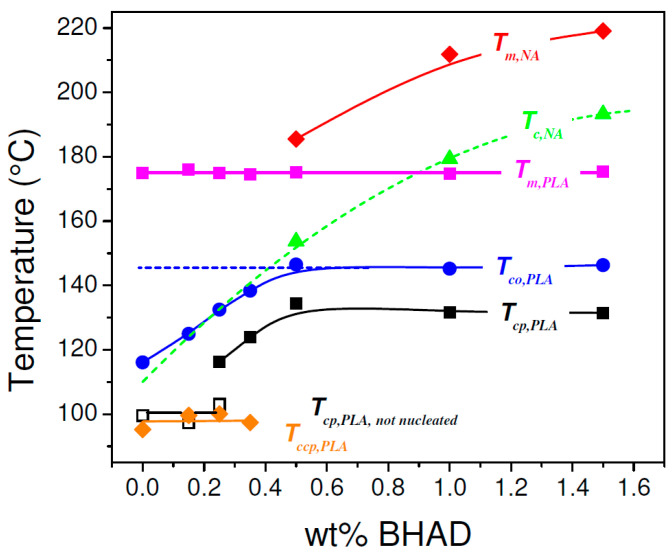
Transition temperatures as function of the BHAD wt% in PLA-1 during cooling and subsequent heating at 10 °C/min: $T_{c,NA}$ (green triangles), $T_{co,PLA}$ (blue circles), $T_{cp,PLA}$ (black squares, open: not nucleated by BHAD, closed: nucleated by BHAD), $T_{ccp,PLA}$ (orange diamond), $T_{m,PLA}$ (magenta squares), and $T_{m,NA}$ (red diamonds). Full lines are guides to the eye using B-splines, except for $T_{m,PLA}$, $T_{cp,PLA}$ (not nucleated by BHA), and $T_{ccp,PLA}$ for which constant trend lines were used. The green dashed curve is a second order polynomial extrapolating $T_{c,NA}$ towards a lower BHAD wt%. This curve intersects the blue dashed extrapolation of the constant part in the $T_{co,PLA}$ evolution close to a BHAD content of 0.4 wt%.

In Figure 5, seven relevant transition temperatures are highlighted: the crystallization peak temperature of the nucleating agent BHAD ($T_{c,NA}$, green triangle), the PLA-1 crystallization onset ($T_{co,PLA}$, blue circle) and peak temperatures during cooling ($T_{cp,PLA}$, open and closed black squares), the PLA-1 cold crystallization peak temperature during heating ($T_{ccp,PLA}$, orange diamond), the PLA-1 melting peak temperature ($T_{m,PLA}$, magenta square), and the nucleating agent melting peak temperature ($T_{m,NA}$, red diamond). $T_{co,PLA}$ was defined as the temperature where the transition enthalpy, $h_{a}\left(T\right)-h\left(T\right)$ (Figure 5, middle panels), reached 0.001 J g^−1^. These transition temperatures are shown in Figure 7 as a function of the BHAD wt% in PLA-1.

$T_{co,PLA}$ increases with increasing wt% BHAD up to reaching 0.5 wt%, beyond which it remains constant. For 0.5 wt% BHAD or more, BHAD crystallization was detected with a peak at $T_{c,NA}$ (Figure 5). In these cases, PLA-1 crystallization is triggered in the melt, containing BHAD crystallites. Optical microscopy demonstrates that BHAD crystals nucleate the PLA-1 crystallization.

**Figure 8:**
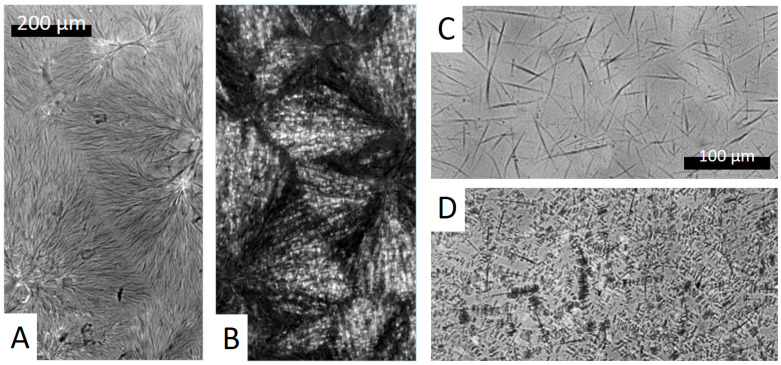
Optical microscopy images collected with (panel **B**) and without (panels **A**–**D**) crossed polarizers. The panels **A** and **B** relate to PLA-1 with 1 wt% BHAD at 160 °C (**A**) and 140 °C (**B**) while cooling at 10 °C/min from 230 °C. The panels **C** and **D** relate to PLA-1 with 0.5 wt% BHAD at 150 °C (**C**) and 126 °C (**D**) during cooling at 10 °C/min. The scale bar in panel **A** also holds for panel **B** and the scale bar in panel **C** also for panel **D**.

BHAD creates spherulitically arranged needle-like crystallites when PLA-1 with 1 wt% BHAD (Figure 8A) is cooled from a high temperature melt at 10 °C/min to $T_{c,NA}$. Above that temperature, BHAD and PLA-1 are homogeneously mixed in the melt. The sample with 1.5 wt% BHAD has similar BHAD spherulites but with a higher needle density. When cooled further below $T_{co,PLA}$, PLA-1 epitaxially crystallizes onto the BHAD crystallites, forming shish-kebab structures, with BHAD crystals being the shish and PLA-1 lamellae as the kebabs. These structures are spatially arranged according to the BHAD crystal template (Figure 8B). Such morphologies have also been observed for BHAD in combination with another polyester [52].

The shish-kebab nature of the epitaxial PLA-1 crystallization is clearly seen in Figure 8D. This picture was collected just below $T_{co,PLA}$ for PLA-1 with 0.5 wt% BHAD upon cooling at 10 °C/min. The corresponding naked BHAD crystal needles are shown in Figure 8C. The latter image was taken in between $T_{c,NA}$ and $T_{co,PLA}$. Isolated BHAD needles are created at that composition, rather than spherulitic assemblies. The lack of connectivity in this case implies that each BHAD crystal sprouted independently. This seems a logical consequence of the lower BHAD wt%.

**Figure 9:**
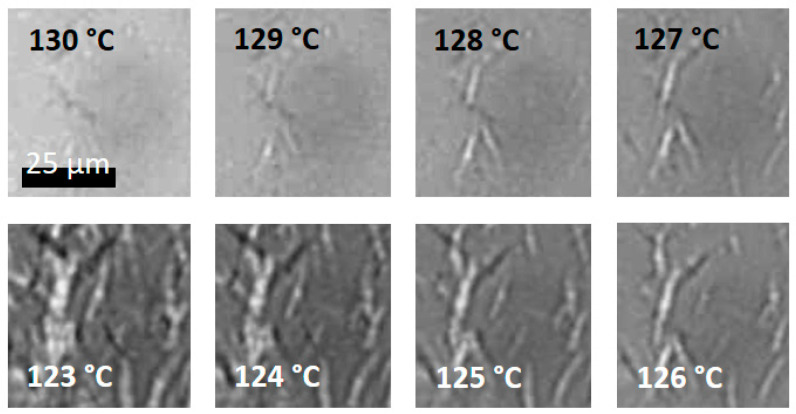
Optical microscopy images of PLA-1 with 0.25 wt% BHAD from the onset of crystallization at 130 °C down to 123 °C. A group of individual BHAD crystallites is generated and rather instantaneously overgrown by PLA-1 crystallites.

Although no separate BHAD crystallization peak could be observed for the samples with less than 0.5 wt% BHAD, BHAD still nucleates PLA-1. The crystallization and melting temperatures of BHAD in PLA-1 are depressed, as commonly observed for melt miscible systems [53,54]. Indeed, $T_{m,NA}$ and $T_{c,NA}$ for pure BHAD are found to be 240.9 °C and 209.1 °C, respectively [52], which is much higher than $T_{m,NA}$ and $T_{c,NA}$ in blends with PLA-1 (Figure 7). The decreasing trend of $T_{c,NA}$ with decreasing BHAD wt% was extrapolated using a second order polynomial (the green dashed curve in Figure 7). Clearly, the extrapolated $T_{c,NA}$ values for the samples with less than 0.5 wt% BHAD coincide with $T_{co,PLA}$, suggesting that PLA-1 crystallizes as soon as BHAD crystallites are formed. This effect hides $T_{c,NA}$ in DSC. The BHAD exothermic transition furthermore escapes the observation because of being very small for these low BHAD concentrations. Nevertheless, optical microscopy is able to capture this overlapping BHAD and BHAD-nucleated PLA-1 crystallization as illustrated in Figure 9 for PLA-1 with 0.25 wt% BHAD. The crystalline BHAD needles appear isolated, and their concentration and length is much reduced compared to the material with 0.5 wt% BHAD (Figure 8C). This seems a logical consequence of the further reduced BHAD concentration.

In principle, each NA has a specific temperature at which it induces heterogeneous nucleation. This temperature depends on the actual decrease of the energy barrier toward polymer nucleation that a given NA can realize. $T_{cp,PLA}$ depends on the BHAD wt% up to reaching 0.5 wt% (Figure 7), not because of (unrealistic) concentration dependent differences in BHAD surface characteristics but simply because it needs to be crystalline to be effective. Once BHAD is in its crystalline state, it rather efficiently nucleates PLA crystallization, leading to a characteristic $T_{co,PLA}$ of 146 °C, calculated as the average $T_{co,PLA}$ of PLA-1 with 0.5, 1, and 1.5 wt% BHAD. Extrapolating this temperature to lower BHAD wt% (the blue line in Figure 7) leads to an intersection with the green dashed curve at approximately 0.4 wt% BHAD. This is the minimum BHAD wt% needed to ensure BHAD crystals for a most efficient PLA-1 crystallization, i.e., starting at 146 °C when cooled at 10 °C/min. Feng et al. determined the actual nucleation efficiency, NE%, of BHAD by means of DSC [55], using the approach suggested by Fillon et al. [56]. They concluded that the NE% of BHAD at 0.5 wt% in PLA is 50%, which is twice as high as the value for talc.

The evolution of $T_{cp,PLA}$ follows the trend of $T_{co,PLA}$, but is roughly 15 °C lower for samples containing at least 0.35 wt% BHAD (the black squares in Figure 7). At 0.25 wt% BHAD, a second crystallization peak at lower temperatures appears. This peak (open black squares in Figure 7) is the only one remaining at even lower BHAD contents, down to pure PLA-1. The position of this peak is rather constant and—as discussed earlier—is most likely due to spontaneously nucleated PLA-1 crystallization without BHAD interference. This type of crystallization continues during cold crystallization upon heating. This event peaks at a $T_{cp,PLA}$ which is rather independent from the BHAD wt% (orange diamonds in Figure 7) and coincides rather well with the constant $T_{cp,PLA}$ values of spontaneously nucleated PLA-1.

$T_{m,PLA}$ is rather constant whereas $T_{m,NA}$ decreases. Such behavior is expected for monotectic systems [53], with the eutectic composition occurring at the intersection of the melting peaks, which upon extrapolation is expected to occur at approximately 0.35 wt% BHAD. At higher BHAD wt%, $T_{m,PLA}$ indeed should remain constant at the eutectic value. At lower BHDA wt%, a depression of $T_{m,PLA}$ is expected [53], which in this case does not seem to exceed the experimental uncertainty on $T_{m,PLA}$.

### 3.3. Binary Mixtures of PLA-2 and PLA-1 with the Plasticizer PEG 1000

The efficiency of a plasticizer in a polymer blend can be accessed via the blend glass transition temperature, $T_{g,blend}$, which in the present study was obtained from the inflection point at the $c_{p}(T)$ jump. Miscible plasticizers, such as PEG, featuring a lower glass transition temperature, $T_{g,PEG}$, than the PLA glass transition temperature, $T_{g,PLA}$, are expected to lower $T_{g,blend}$ with increasing plasticizer mass fraction, $w_{PEG}$, according to the Fox equation [57]:

(9) $$\frac{1}{T_{g,blend}}=\frac{\left(1-w_{PEG}\right)}{T_{g,PLA}}+\frac{w_{PEG}}{T_{g,PEG}}$$

or the Gordon-Taylor equation [58]:

(10) $$T_{g,blend}=\frac{\left(1-w_{PEG}\right)T_{g,PLA}+K_{GT}w_{PEG}T_{g,PEG}}{\left(1-w_{PEG}\right)+K_{GT}w_{PEG}}$$

**Figure 10:**
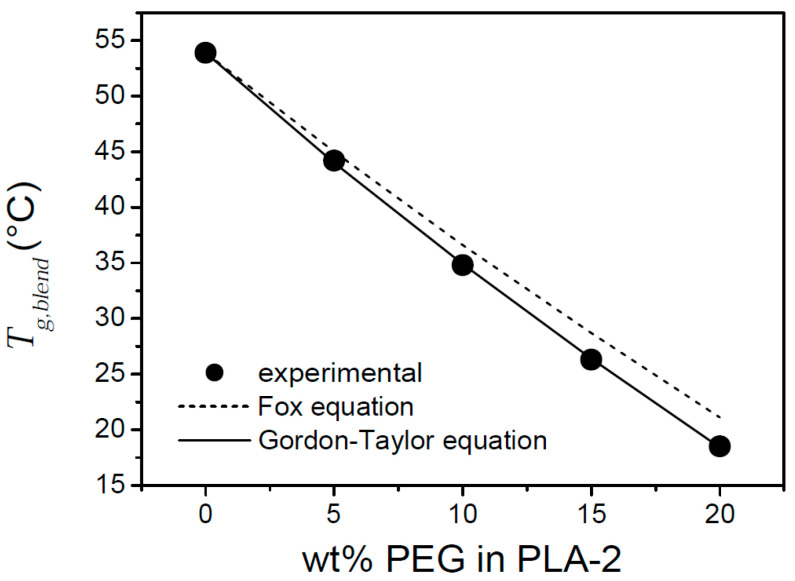
$T_{g,blend}$ as a function of the PEG wt% in PLA-2.

In both equations, absolute temperatures are used. In Figure 10, $T_{g,blend}$ is depicted as a function of $w_{PEG}$ (expressed as wt%) in blends of PEG 1000 with amorphous PLA-2. These values were obtained during cooling at 10 °C/min. A decrease from 53.9 °C for pure PLA-2 to 18.5 °C was found by increasing $w_{PEG}$ up to 20 wt%. This decrease reveals a good plasticizing efficiency. A theoretical prediction of $T_{g,blend}$ via Equations (9) and (10) was made, making use of 53.9 °C for $T_{g,PLA}$ and −63 °C for $T_{g,PEG}$. The latter value was obtained with a Flash DSC 1 (Mettler Toledo), applying a cooling rate of 10³ °C/s to prevent PEG crystallization prior to vitrification (M. Colaers, unpublished results). The prediction based on Equation (10) covered the experimental data adequately, the one based on Equation (9) did not (Figure 10). The interaction constant, $K_{GT}$, in Equation (10) was obtained through fitting and equaled 1.742.

Plasticizers can promote guest polymer crystallizability by increasing the temperature window for crystallization by decreasing $T_{g}$, as well as by increasing the crystallization rate through an increase of the chain mobility [5,32]. Such effects prevail during crystallization at high supercooling (low temperatures). In principle, the crystal growth rate can also decrease with increasing plasticizer content when crystallized at rather low supercooling (high temperature) because plasticizer addition progressively lowers the polymer equilibrium melting point, $T_{m}^{0}$ [59,60]. Crystallization at a given temperature thus happens at a reduced supercooling with respect to $T_{m}^{0}$ and should reduce the nucleation controlled crystallization rate, unless this effect is overcompensated by the enhanced mobility. Under the current conditions (cooling and heating at 10 °C/min), crystallization always happens below 140 °C. At this temperature, and consequently at any lower temperature, the mobility-related crystallization enhancement prevails, as demonstrated in the next paragraph.

**Figure 11:**
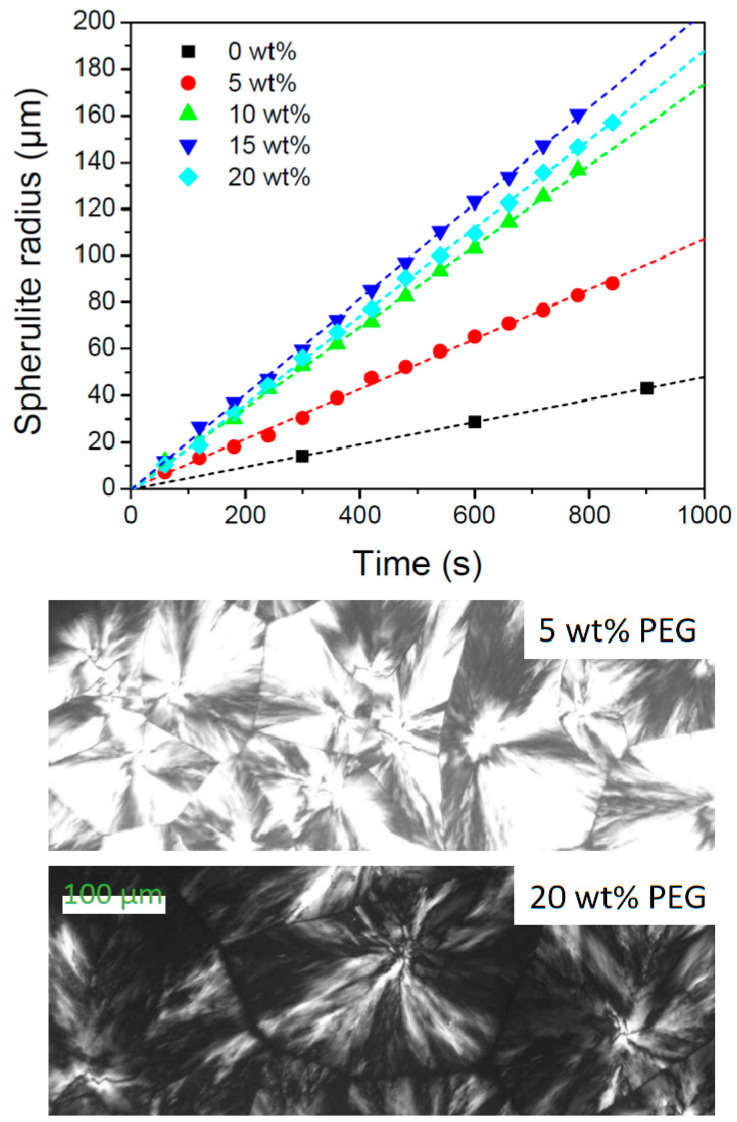
(**Top**): The spherulite radius measured by polarized optical microscopy (POM) for PLA-1 with various PEG amounts during isothermal crystallization at 140 °C. Middle: spherulites at the end of the POM experiment for the sample with 5 wt% PEG. (**Bottom**): POM image at the end of the crystallization for the 20 wt% PEG sample exhibiting non-birefringent (black) PEG accumulations at the spherulite borders.

PLA-1 samples with various PEG amounts were isothermally crystallized at 140 °C after cooling from 200 °C while monitoring the spherulite growth rate. The result is depicted in Figure 11. The related growth rates for 0, 5, 10, 15, and 20 wt% PEG are 0.05, 0.10, 0.17, 0.21, and 0.19 µm/s, respectively. For this set of experiments, the actual PEG content was not checked by NMR. Therefore, nominal PEG concentrations are displayed. The growth rates thus increase with increasing PEG content, except for the 20 wt% PEG sample for which the trend inverts. The lower growth rate for this sample can in principle result from a reduced driving force for crystallization due to a decreased supercooling [59]. However, it will be shown further down in this section that the PLA-1 melting point drops with increasing PEG wt%, but the $T_{m,PLA}$ reduction—which is assumed to reflect the relative $T_{m}^{0}$ evolution—is too small to induce such marked effects. Alternatively, this rate swap can be linked to a changeover in PEG segregation mechanism while PLA-1 is crystallizing. The spherulites of all samples, except for the 20 wt% PEG sample, ultimately collide at the end of crystallization, as illustrated for the 5 wt% PEG sample in the middle panel of Figure 11. In contrast, the spherulite borders for the 20 wt% sample exhibit dark, non-birefringent zones, suggesting PEG accumulation, which should hinder growth as this shields the growth front from the PLA-rich melt. In principle, a progressive accumulation should lead to nonlinear spherulitic growth [61], which was not observed. Thus, it seems that a constant accumulation is rapidly established at the growth front and pushed forward. Such a steady state implies a concomitant PEG deposition within the spherulite between crystalline lamellae (inter-lamellar segregation) or lamellar bundles (inter-fibrillar segregation). This intraspherulitic segregation dominates for all other compositions as no PEG accumulation at the spherulite borders was observed.

**Figure 12:**
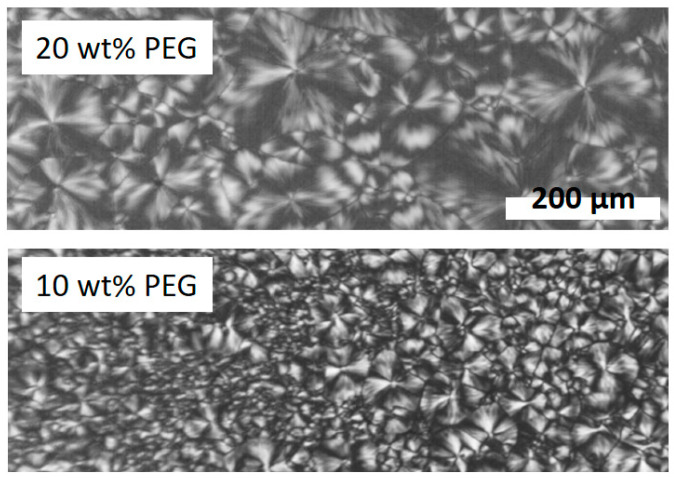
Spherulites at room temperature after cooling PLA-1 with 20 and 10 wt% PEG at 10 °C/min from 200 °C.

The observed interspherulitic PEG accumulation for the 20 wt% PEG sample conflicts with earlier work on PLA plasticized with even higher PEG 400 contents and for which no interspherulitic PEG segregation was found when crystallized at 110 °C [59]. Furthermore, Figure 12 shows impinging spherulites at 20 °C of samples with 20 and 10 wt% PEG after cooling from 200 °C at 10 °C/min without indications of interspherulitic PEG segregation. It will be shown below that crystallization under these conditions peaks at about 100 °C. It thus seems that the interspherulitic segregation for the 20 wt% sample at 140 °C is suppressed when crystallized at lower temperatures. This makes sense because the ratio of the crystal growth rate over the impurity (i.e., the plasticizer) segregation rate decreases at lower temperatures and should lead to more PEG trapped inside the spherulites. When interspherulitic segregation vanishes, the inversion in the growth rate trend will likely disappear as well. Therefore, for the PEG concentrations and crystallization conditions relevant to the present study (i.e., cooling at 10 °C/min), very likely the crystal growth rates just increase with increasing PEG content.

**Figure 13:**
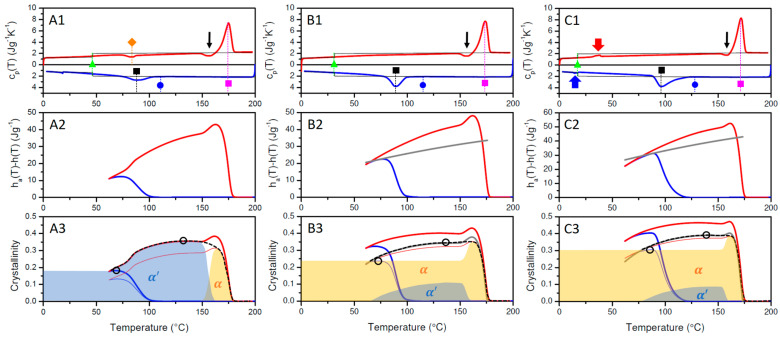
DSC measurement of PLA-1 with 4.75 (**A**), 9.75 (**B**), and 16.63 (**C**) w% PEG during cooling (blue curves) and subsequent heating (red curves). The panels **1**, **2**, and **3** respectively cover the $c_{p}(T)$, transition enthalpy, and crystallinity (thick and thin lines for treatments in terms of α′ and α crystals, respectively). The thin black curves in the panels **1** are reference $c_{p}\left(T\right)$ values for amorphous PLA given by the Equations (2) and (3) and a changeover at a calculated $T_{g,blend}$. The black arrows point at the exothermic conversion from the α′ to the α phase. The blue and red arrows in panel (**C1**) highlight signals related to the crystallization and melting of PEG, respectively. Certain peak and onset temperatures of thermal transitions are highlighted using vertical lines with symbols: $T_{g,blend}$ (green triangles), $T_{co,PLA}$ (blue circles), $T_{cp,PLA}$ (black squares), $T_{ccp,PLA}$ (orange diamond), and $T_{m,PLA}$ (magenta squares). The grey line in the panels (**B2**,**C2**) are calculated transition enthalpies, assuming a constant α crystallinity at the level reached during preceding cooling. The dashed black curves in the bottom panels represent the overall crystallinity evolution during heating, which is to be interpreted as a superposition of α′ (blue area) and α (yellow area) crystallinity. The thick grey curves in the panels (**B3**,**C3**) illustrate evolutions of mixed of α′ and α crystallinities for the heating run. The circled data points highlight the maximum crystallinity values reached during cooling and heating.

Figure 13 illustrates the DSC-based crystallization and melting behavior for PLA-1 with 4.75 (A-panels), 9.75 (B-panels), and 16.63 wt% (C-panels) PEG during cooling and subsequent heating at 10 °C/min. All $c_{p}(T)$ cooling curves clearly exhibit crystallization exothermic signals during cooling with onsets at the blue circles and peaks at the black squares (Figure 13(A1,B1,C1)). Onsets are again placed at temperatures where the transition enthalpy, $h_{a}\left(T\right)-h\left(T\right)$ (Figure 13, middle panels), reached 0.001 J g^−1^. The areas of the exothermic peaks increase with increasing PEG content. This is reflected in the higher maximum transition enthalpies reached during cooling (blue curves, Figure 13, middle panels). The addition of PEG thus stimulates crystallization during cooling. Note that the transition enthalpies are related to PLA-1 only as the PEG fraction is taken into account in Equation (5). Furthermore, $c_{pl,PLA-PEG}(T)$ (Equation (3)) rather than $c_{pl,PLA}\left(T\right)$ (Equation (1)) was used in Equation (5).

Further cooling led to vitrification at $T_{g,blend}$, of which the temperatures are highlighted using green triangles in Figure 13. At these temperatures, the amorphous reference $c_{p}(T)$ (given by the thin black lines) steps down from the liquid to the glassy state, following values given by the Equations (3) and (2), respectively, and making use of the PEG content, $w_{PEG}$, in Equation (3). The $T_{g,blend}$ values were calculated using Equation (10), rather than experimentally determined. However, it is not appropriate to use $w_{PEG}$ in this equation. PLA-1 crystallization leads to a PEG enrichment in the remaining amorphous phase with a mass fraction, $w_{PEG,enriched}$, given by:

(11) $$w_{PEG,enriched}=\frac{w_{PEG}}{w_{PEG}+\left(1-w_{PEG}\right)\left(1-w_{c}\left(T_{g,blend}\right)\right)}$$

with $w_{c}\left(T_{g,blend}\right)$ being the PLA crystallinity at $T_{g,blend}$. Therefore, $w_{PEG,enriched}$ was used instead of the overall $w_{PEG}$ in Equation (10) to calculate $T_{g,blend}$. The relevant crystallinity for this calculation is the circled value in the cooling runs of the bottom panels of Figure 13. A full discussion on the crystallinity follows further down in this section.

Interestingly, the 16.63 wt% PEG sample exhibits a small exothermic peak just below $T_{g,blend}$ (blue arrow in panel C1, Figure 13), which is attributed to the crystallization of segregated PEG [40]. As Figure 12 does not provide any evidence for interspherulitic segregation, this crystallizing PEG most likely stems from PEG segregated at the interfibrillar level.

Subsequent heating beyond $T_{g,blend}$ led to a PEG crystal melting peak for the sample with 16.63 wt% PEG (red arrow in panel C1) and a cold crystallization peak (orange diamond in panel A1) for the sample with 4.75 wt% PEG. No cold crystallization peaks are observed for the 9.75 and 16.63 wt% PEG samples (panels B1 and C1). All samples display an exothermic signal due to an α′ − α transition (black arrows) prior to full melting with an endothermic peak at the magenta squares in the top panels of Figure 12.

**Figure 14:**
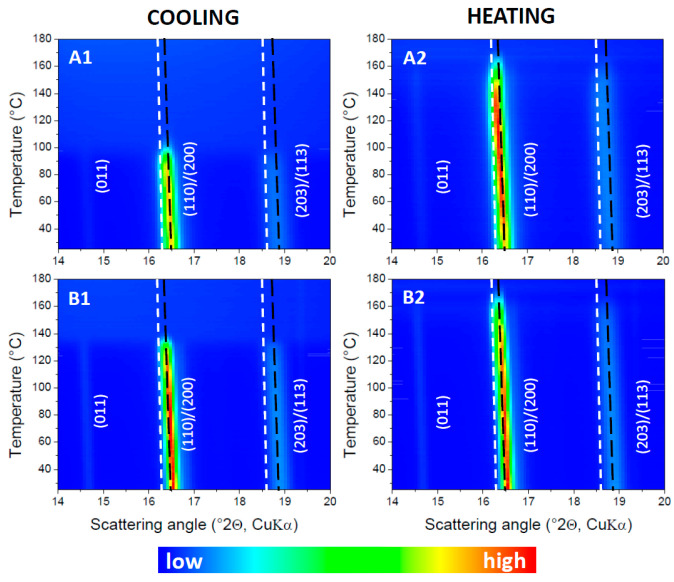
WAXD patterns during cooling (**A1**,**B1** panels) and subsequent heating (**A2**,**B2** panels) at 10 °C/min for PLA-1 with 15 wt% PEG ((**A**)-panels) and PLA-1 with 10 wt% PEG and 1 wt% BHAD ((**B**)-panels). The patterns are top viewed with color coded intensities from low (blue) to high (red) intensities according to the color scale at the bottom. Numbers in between brackets are miler indices of pertinent reflections. The dashed lines mark out the tracks of the mixed (110)/(200) and (203)/(113) reflections expected for the α′ phase in white and for the α phase in black [7].

The observation of an α′ − α transition for each sample in Figure 13 implies that at least a fraction of the PLA-1 crystallinity should be of the α′ type, irrespective of the PEG content. Synchrotron WAXD experiments were conducted on PLA-1 with 15 wt% PEG. During cooling, all crystals are of the α type, as the experimental diagnostic reflections follow the reference α tracks, i.e., the black dashed lines in Figure 14(A1). However, during subsequent heating, the experimental α reflections develop a shoulder towards the α′ tracks (the white dashed lines), suggesting that α′ crystals are formed (Figure 14(A2)). At the highest temperatures, where the reflections decrease as a result of melting, the reflections again fully coincide with the α tracks. This observation was translated to the DSC crystallinity analysis depicted in Figure 13(C3) for the sample with 16.63 wt% PEG. A procedure similar to that used for the sample with 0.35 wt% BHAD was followed. More specifically, the crystallinity developed during cooling was associated with α crystals whereas additional crystallinity during heating was assigned to α′ crystals. The overall crystallinity evolution during heating is represented using a black dashed line, and includes a transfer from the grey, representing a mixed α and α′ crystallinity, to the thin red curve, representing pure α crystals. This transfer is horizontal, implying that the conversion from α′ (reaching a maximum weight fraction of only 0.098) to α is quantitative. A similar analysis was performed on PLA-1 with 9.75 and 13.75 wt% PEG. The outcome for the 9.75 wt% PEG sample is shown in Figure 13B. For both samples, the α′ − α conversion was also rather quantitative. In other words, all α′ crystal convert to α crystals prior to melting.

The outlined DSC phase assignment surely makes sense for the 13.75 and 16.63 wt% PEG sample, given the proximity in composition with the 15 wt% PEG sample for which there is WAXD evidence. The assignment for the 9.75 wt% PEG sample seems justified because the outcome is very similar in nature to that of the 13.75 and 16.63 wt% PEG samples: the α′ crystals during heating are generated rather gradually over a wide temperature range.

The behavior of the 4.75 wt% PEG sample is, however, different (Figure 13A). The crystallization during cooling is arrested by vitrification and resumed during subsequent heating in a cold crystallization event, peaking at the orange diamond in Figure 13(A1). Such behavior is not seen for the samples with higher PEG contents but resembles that of pure PLA-1. Therefore, the data were interpreted in the same way as for pure PLA-1, involving α′ crystallization during cooling as well as during subsequent heating and an α′ − α transition at the highest temperatures.

**Figure 15:**
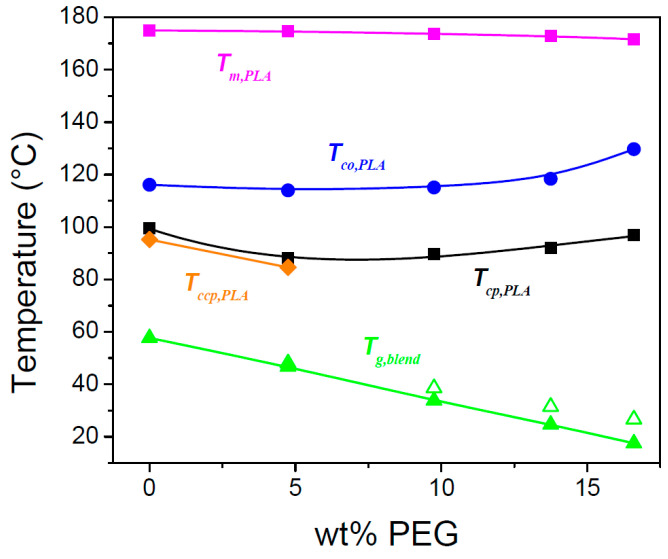
Transition temperatures as a function of the PEG wt% (NMR verified) in PLA-1 during cooling and heating at 10 °C/min: $T_{g,blend}$ (green triangles, closed: taking into account the crystallization induced PEG enrichment, open: without doing so), $T_{co,PLA}$ (blue circles), $T_{cp,PLA}$ (black squares), $T_{ccp,PLA}$ (orange diamond), and $T_{m,PLA}$ (magenta squares). Full lines are guides to the eye using B-splines. Part of the input to this Figure can be found in Figure 13, using corresponding symbols.

As mentioned earlier, the actual $T_{g,blend}$ depends on the PEG amount, but it is also affected by the crystallinity reached prior to vitrification, as this leads to PEG enrichment. Figure 15 demonstrates that $T_{g,blend}$, based on Equation (10) and the overall $w_{PEG}$ (open green symbols), decreases with increasing PEG content, but that it does so more strongly when taking the PEG enrichment into account, $w_{PEG,enriched}$ (closed green symbols).

Figure 15 also reveals that $T_{co,PLA}$ (blue circles) for the 4.75 wt% PEG sample is identical to that of pure PLA-1 but that $T_{cp,PLA}$ (black squares) shifts to lower temperatures in parallel with the decrease of $T_{g,blend}$. Crystallization in the 4.75 wt% PEG sample thus starts like in pure PLA-1 but progresses further towards lower temperatures because of the reduced $T_{g,blend}$. This illustrates the impact of increasing the temperature window for crystallization by decreasing $T_{g}$. Similarly, cold crystallization during heating for the 4.75 wt% PEG sample is permitted at a lower temperature than in pure PLA-1 (the orange diamonds in Figure 15) because devitrification happens earlier for the PEG containing sample.

**Figure 16:**
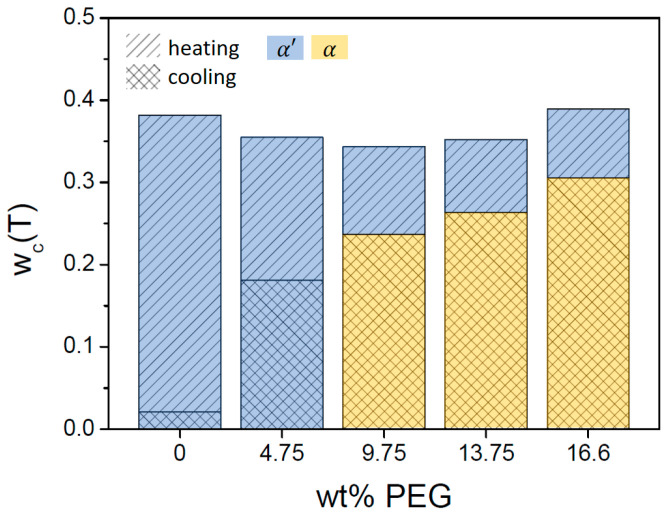
Mass fraction crystallinities reached after cooling at 10 °C/min (crosshatched parts) and the further increase realized in the subsequent heating run at the same rate (diagonally hatched parts) for PLA-1 as a function of the (NMR verified) PEG wt%. The total bar height represents the sum of contributions during cooling and heating. The crystal form (α′ in blue, α in yellow, and mixed forms in greenish shading) is indicated. Errors on crystalline fractions are ±0.01 based on the standard deviation of repeated measurements. This Figure contains the data marked with the open black circles in the Figure 2 and Figure 13.

The crystallization during cooling for the samples with more than 5 wt% PEG is not arrested by vitrification because $T_{g,blend}$ decreased rather strongly (Figure 15). At first sight, it seems that crystallization is complete for these compositions, since cold crystallization is absent and because space filling spherulites are seen after cooling (Figure 12). However, although the primary crystallization is complete, this is not so for secondary (intraspherulitic) crystallization. The maximum crystallinities reached during cooling and subsequent heating for PLA-1 in the presence of PEG are displayed in Figure 16. Clearly, although adding PEG simulates crystallization during cooling, the values reached during subsequent heating (0.35, 0.34, and 0.35 for the samples with 4.75, 9.75, and 13.75 wt% PEG, respectively) remain below the value reached for pure PLA-1 (0.38), except for the 16.6 wt% PEG sample, for which the ultimate value (0.39) is identical. For most compositions, PEG thus prevents reaching the ultimate PLA-1 crystallinity during heating, likely because uncrystallized PLA remains highly diluted in PEG enriched intraspherulitic pockets. Part of this diluted PLA seems to be able to crystallize into metastable α′ crystals during subsequent heating after stimulated nucleation by the excursion below $T_{g,blend}$.

This reduced tendency to crystallize seems to be overcome in the sample with 16.6 wt% PEG. Figure 15 illustrates that $T_{co,PLA}$ in this sample occurs at a significantly higher temperature compared to the other samples, implying that PEG segregation at the creation of the first crystals is very efficient (cfr. the discussion related to the Figure 11 and Figure 12) and locally leads to a high PLA-1 crystallinity during that part of the cooling process. It also leads to rather pure PEG pockets which at low temperature crystallize themselves (Figure 13). The PEG segregation must, however, be rather inhomogeneous because $T_{g,blend}$ based on $w_{PEG,enriched}$ occurs at a temperature just above the PEG crystallization peak revealed in Figure 13(C1). Vitrification in the PEG enriched inclusions should prevent PEG crystallization, unless the PEG is not evenly distributed. PEG crystallization can happen in the earliest formed, more strongly enriched zones whereas the later formed, less enriched zones should vitrify prior to reaching the calculated $T_{g,blend}$. Figure 13(C1) shows that the PLA-1 crystallization peak is very asymmetric. Although it starts at a high temperature ($T_{co,PLA}$ at the blue circle), it only peaks ($T_{cp,PLA}$ at the black square) as low as the crystallization peak of the sample with 9.75 wt% PEG (Figure 13(B1)). In that regime, segregation should be less efficient and not conducive to PEG crystallization but permitting for additional (secondary) PLA-1 α′ crystallinity upon heating. Note that $T_{m,PLA}$ (magenta squares) in Figure 15 slightly decreases with increasing PEG wt%, as expected on thermodynamic grounds [59,60]. The trends for $T_{co,PLA}$ (blue circles) and $T_{cp,PLA}$ (black squares) are opposite, implying that the crystallization stimulating effect of the PEG-induced enhanced mobility prevails.

The conclusion that PEG addition leads to incomplete crystallization (except for the sample with 16.6 wt% PEG) relies on the phase assignment. Associating the transition enthalpy during cooling to α′ crystallinity instead of α crystallinity would lead to total crystallinities exceeding that of pure PLA-1 for the 9.75, 13.75, and 16.6 wt% PEG samples. Such an assignment would be incorrect, based on the WAXD data. Therefore, earlier claims on PEG related DSC-based crystallinity evolutions in which polymorphism was neglected, should perhaps be reconsidered [34,40]. On the other hand, the crystallinity of the sample with 16.6 wt% PEG reaches the level of pure PLA-1 with part of the crystalline fraction being of the α form under the current conditions of cooling and heating at 10 °C/min. This observation was attributed to partial crystallization at high temperatures where PEG segregation is outspoken. Following this line of thinking, it should be possible to boost the material crystallinity of all PEG containing samples beyond that of pure PLA-1 by conducting crystallization isothermally at high temperatures. Alternatively, and industrially more relevant, one may try to shift the crystallization to higher temperatures by facilitating primary nucleation, i.e., by using a nucleating agent in conjunction with PEG. This is the approach followed in this work, as outlined in Section 4.

A final remark in the current section relates to polymorphism. $T_{cp,PLA}$ (black squares in Figure 15) for all samples with more than 5 wt% PEG falls below 100 °C. At such a low temperature, pure PLA is expected to crystallize in the α′ form [7]. As α crystals are formed instead, it seems that PEG and maybe plasticizers in general, are able to shift the temperature for crystallization in the α form downwards. This theme deserves research attention in the future, as, to the best of our knowledge, no systematic studies on this aspect are available in the literature.

### 3.4. Ternary Mixtures of PLA-1, BHAD, and PEG 1000

**Figure 17:**
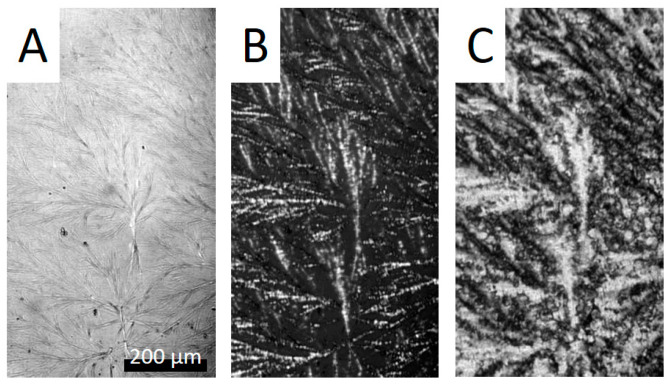
Optical microscopy images of a material containing 90 wt% PLA-1 and 10 wt% PEG to which 1 wt% BHAD was added. Images were collected with (**B**,**C**) and without (**A**) crossed polarizers during cooling at 10 °C/min from 230 °C at 161 °C (panel **A**), 140 °C (**B**), and 20 °C (**C**). The scale bar in panel **A** also holds for panels (**B**,**C**).

This section discusses materials based on a blend of 90 wt% PLA-1 with 10 wt% PEG. This blend is further mixed with 0.5, 1.0, or 1.5 wt% BHAD. In Section 3.2, it was demonstrated that the addition of BHAD to pure PLA-1 at such BHAD concentrations leads to BHAD crystallization of which the crystals during further cooling nucleate PLA-1 crystallization. A similar behavior is observed when the matrix contains 10 wt% PEG, as illustrated in Figure 17 for the case with 1.0 wt% BHAD. A crystalline BHAD skeleton is created at high temperature (panel A, 161 °C), which upon further cooling (panel B, 145 °C and panel C, 20 °C) is epitaxially overgrown by PLA-1 crystals. The BHAD crystal network seems to be somewhat less dense when PEG is present (i.e., less crystals per unit of volume) compared to when only PLA-1 is used but otherwise the behavior is very comparable. This also holds for the systems with 0.5 and 1.5 wt% BHAD. Furthermore, note that it was much harder to visualize the BHAD crystals if 10 wt% PEG was present. The contrast of the picture in Figure 17A needed to be enhanced for better visualization. It cannot be excluded that the refractive index of the mixed PLA-1/PEG matrix more closely matches that of the BHAD crystals by which they tend to escape observation. Their presence is, however, clearly revealed once covered by PLA-1 crystallites.

The WAXD data in Figure 14(B1,B2) reveal that PLA-1 in the material with 10 wt% PEG and 1 wt% BHAD crystallizes as α crystals and that the polymorphism was not affected upon heating. Indeed, the experimental reflections faithfully follow the α reference tracks at all temperatures. A preference for α crystallinity was also observed for 1 wt% BHAD in the absence of PEG (Section 3.2). At 10 wt% PEG without BHAD, cooling induced α crystallinity at the primary crystallization during cooling, but additional secondary crystallization during heating in the PEG enriched pockets was of the α′ form (Section 3.3). With this information, the calorimetric data for the three components systems can adequately be interpreted.

**Figure 18:**
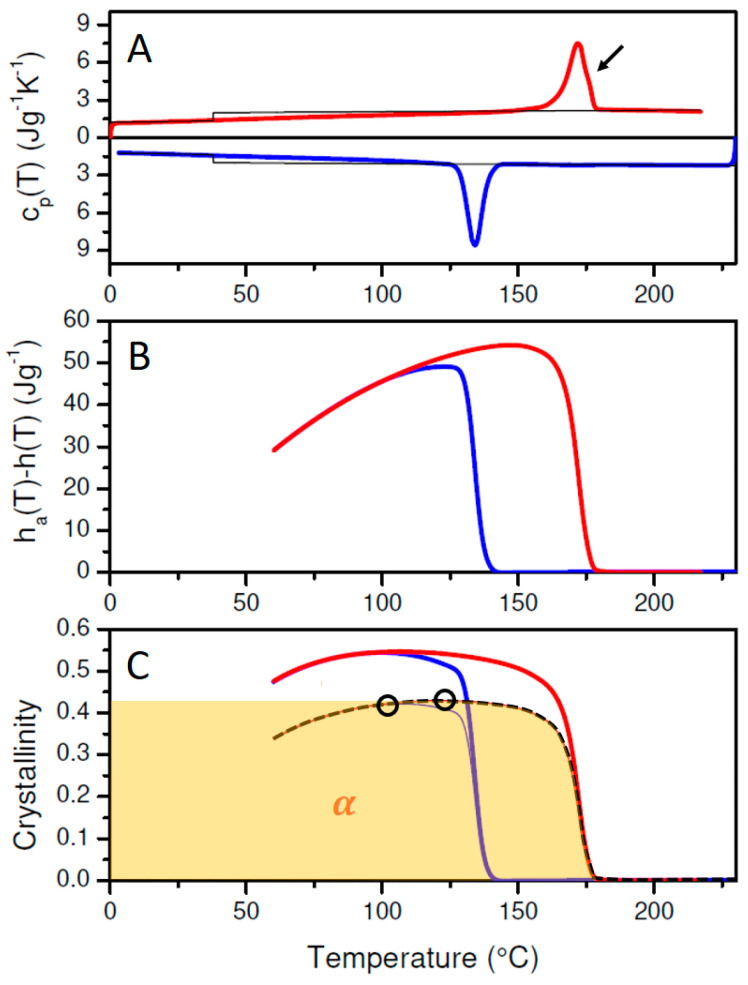
(**A**): DSC cooling (blue) and subsequent heating (red) run on PLA-1 with 10 wt% PEG and 1 wt% BHAD at 10 °C/min between 230 and 0 °C at 10 °C/min. The thin black curves are reference $c_{p}\left(T\right)$ values for amorphous PLA given by the Equations (2) and (3) and a changeover at a calculated $T_{g,blend}$. The arrow points at the high temperature shoulder, as a result of melting recrystallization from α to α PLA. (**B**): transition enthalpy values during cooling (blue) and heating (red) derived from the $c_{p}\left(T\right)$ values; (**C**): $c_{p}\left(T\right)$ based crystallinity values, assuming α′ (thick curves) or α (thin curves) crystallinity during cooling (blue) and heating (red). The dashed black curve represents the overall crystallinity evolution during heating, which in this case is fully due to α (yellow area) crystallinity. The circled data points highlight the maximum crystallinity values reached during cooling and heating.

**Figure 19:**
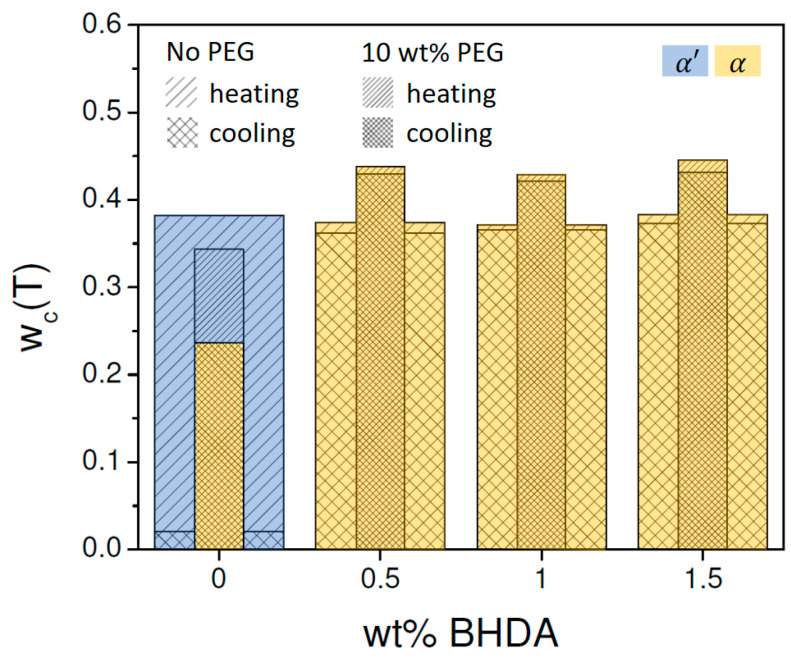
Mass fraction crystallinities reached during cooling at 10 °C/min (crosshatched parts of the bars) and the further increase realized in the subsequent heating run at the same rate (diagonally hatched parts of the bars) for PLA-1 as a function of the BHAD wt%. The total bar height represents the sum of contributions during cooling and heating. The crystal form (α′ in blue, α in yellow) is indicated. The wide and thin bars relate to systems containing pure PLA-1 and PLA-1 combined with 10 wt% PEG, respectively.

Figure 18 displays the DSC-based thermal behavior of the system with 10 wt% PEG and 1 wt% BHAD using the same format as in Figure 2, Figure 5 and Figure 13. The reference $c_{p}\left(T\right)$ curves in Figure 18, panel A were defined as for the systems with PEG (Section 3.2). The calculation of $T_{g,blend}$, i.e., the temperature at which the step in the reference curves occurs, takes the crystallization induced PEG enrichment into account. As done throughout this work, the transition enthalpy was translated into an α′ and α crystallinity, but given the discussion above, only the curves related to the α crystallinity are realistic. The α crystallinity curve during heating is highlighted using a black dashed curve and a yellow shading. An identical analysis was made on 10 wt% PEG systems with 0.5 and 1.5 wt% BHAD. The maximum α crystallinities (i.e., the circled values in Figure 18C for the sample with 1 wt% BHAD) during cooling and subsequent heating are collected in Figure 19.

For pure PLA-1 and PLA-1 combined with 10 wt% PEG, the addition of BHAD leads to α crystallinity throughout. In both cases, crystallization during cooling seems to be complete, i.e., hardly any additional crystallinity is generated during heating (Figure 19). Most strikingly, the maximum crystallinity for the studied BHAD loaded systems with 10 wt% PEG is 0.44 whereas that of the counterparts without PEG is only 0.38 (average values). This is a significant increase. Recall that addition of BHAD (without PEG) brings the crystallinity to the level of pure PLA-1 and that adding 10 wt% PEG (without BHAD) even leads to a reduced crystallinity. Only the combination of BHAD and PEG leads to an increased crystallinity under the applied thermal program (cooling and heating at 10 °C/min).

**Figure 20:**
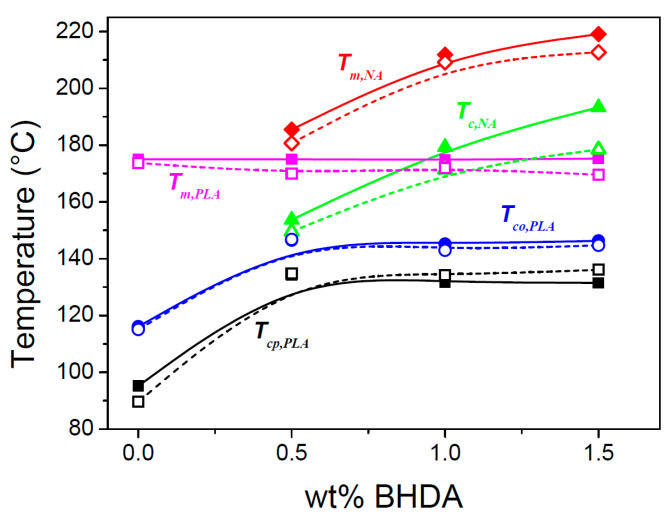
Transition temperatures as a function of the BHAD wt% in pure PLA-1 (closed symbols and full lines) and PLA-1 with 10 wt% PEG (open symbols, dashed lines) during cooling and subsequent heating at 10 °C/min: $T_{c,NA}$ (green triangles), $T_{co,PLA}$ (blue circles), $T_{cp,PLA}$ (black squares), $T_{m,PLA}$ (magenta squares), and $T_{m,NA}$ (red diamonds). Lines are guides to the eye using B-splines.

In Section 3.3, it was anticipated that the full potential of the plasticization enhanced mobility to increase PLA-1 crystallinity could only be reached if the adverse effect of plasticizer dilution could be avoided. Crystallization at high temperatures was suggested as a potential route to realize such conditions. Indeed, Figure 20 demonstrates that $T_{cp,PLA}$ of the blends with 10 wt% PEG (open symbols) is shifted nearly 40 °C upward with the addition of 0.5, 1.0, or 1.5 wt% BHAD, which should promote PEG segregation at the benefit of PLA-1 crystallinity.

Figure 18C hints at a pronounced PEG segregation. The crystallinity curves bend downward to lower crystallinities at low temperatures. This is a methodological artifact related to the involvement of rigid amorphous matter, as explained in Section 2. It also accounts for the (apparent) small crystallinity increase realized during heating, as visualized in Figure 6 and Figure 19 for systems with 0.5 wt% BHAD or more. For the PEG containing systems, these effects suggest a thorough PEG separation; otherwise, the onset of rigid amorphous vitrification (revealed through the artificial downward bending of the crystallinity) would have shifted to lower temperatures by plasticization. In other words, the semicrystalline PLA-1 stacks should be rather pure, and PEG should thus be segregated to an interfibrillar level. In the present case, *interfibrillar* should be read as *inter-shish-kebab*.

In general, the addition of PEG leads to a depression of the BHAD and PLA-1 transition temperatures (Figure 20), except for $T_{cp,PLA}$ for systems with 1.0 and 1.5 wt% BHAD. This is a kinetic effect, illustrating how the high nucleation efficiency of BHAD in conjunction with the increased PEG related mobility and crystal growth rate lead to an efficient crystallization process, translating into a narrow crystallization signal. This swift crystallization leads to a rather high α crystallinity, but on the other hand, the crystals seem to lack perfection, as their melting is accompanied by partial recrystallization and remelting, as revealed by the high temperature shoulder on the PLA-1 melting endotherm. This shoulder is highlighted using an arrow in Figure 18A. Likely, the pronounced nucleation and fast growth leads to frequent crystal collision and hence a lower perfection. This lower perfection leads to a lower $T_{m,PLA}$ and accounts for the apparently stronger PEG-induced $T_{m,PLA}$ depression for systems with BHAD compared to when no BHAD is present (Figure 20).

## 4. Conclusions

The study investigated the crystallinity of Polylactic Acid (PLA) during cooling and heating at 10 °C/min, employing N, N-bis(benzoyl) hexanedioic acid dihydrazide (BHAD) as a nucleating agent and PEG 1000 as a plasticizer. Two grades of PLA, semicrystalline (PLA-1) and amorphous (PLA-2), were analyzed using Differential Scanning Calorimetry (DSC), Wide Angle X-ray Diffraction (WAXD), and Polarized Optical Microscopy (POM). Pure PLA-1 displayed 2% α′ crystallinity during cooling, which increased upon heating to 38% α′ crystallinity due to cold crystallization. PLA-2 remained amorphous, showing only a glass transition.

In binary blends with PLA-1, at least 0.4% BHAD was needed to most efficiently nucleate PLA epitaxially on its needle-like crystallites. Beyond this BHAD content, all PLA-1 crystals during cooling were of the α form and no additional cold crystallization happened during heating. The α crystallinity reached the same level as the α′ crystallinity in pure PLA-1 during heating (38%).

PEG addition above 5 wt% stimulated α crystallization during cooling and additional, more cumbersome intraspherulitic α′ crystallization within PEG enriched regions during heating. The ultimate crystallinity remained limited to 35%, unless very high PEG contents were used. At the highest studied PEG content (16.6 wt%), crystallization happened at high temperatures where PEG segregation is very efficient, leading locally to rather pure PEG and PLA regions instead of PLA partially trapped in PEG enriched regions. At an enhanced segregation, the PLA purity permits reaching a higher crystallinity, which was 38% for the 16.6 wt% PEG sample.

In ternary PLA-1/PEG/BHAD systems, PLA-1 with 10 wt% PEG was complemented with low amounts of BHAD. This BHAD addition shifted the crystallization peak temperature during cooling nearly 40 °C upward compared to when no BHAD was added. Crystallization during cooling was completed and entirely of the α type. No additional crystallinity was generated during heating. Most strikingly, the maximum crystallinity for these ternary systems was 44%.

It seems that PEG addition is needed to enhance the crystallinity by increasing the chain mobility so that chain reconfiguration and disentanglement are promoted. However, this only works efficiently if crystallization happens at high temperatures where PEG segregation is outspoken and leaves rather pure PLA instead of PLA trapped in PEG enriched regions. High temperature crystallization can be triggered by using a nucleating agent like BHAD. Adding BHAD without PEG most likely leads to omnipresent but crowded crystallization with the crystallinity being limited because of an inefficient chain disentanglement.

The present findings contribute to the development of tough, transparent, and highly crystalline PLA materials with enhanced barrier properties. The conclusions primarily rely on DSC analysis, in which PLA’s polymorphism and the temperature dependence of its thermodynamic properties were explicitly considered. This refined approach is essential for accurately interpreting subtle variations in crystallization and melting enthalpies, and for deriving reliable crystallinity values. However, the cooling rate used in this methodological study (10 °C/min) is significantly lower than those typically encountered in industrial processing. Consequently, future research should focus on investigating the effects of more realistic, higher cooling rates on BHAD nucleation efficiency, PEG segregation, and PLA crystallinity, as well as on the resulting mechanical properties and transparency of these promising materials.

## Figures and Tables

**Table 1:** PEG Plasticizer content in (DSC) samples of binary PLA-1/PEG blends determined by H^1^-NMR and compared to the nominal fraction added during blending.

PEG wt% Added	PEG wt% by NMR
5	4.75
10	9.75
15	13.75
20	16.60

## Data Availability

The raw data supporting the conclusions of this article will be made available by the authors on request.
